# Interventions to improve access to cancer care in underserved populations in high income countries: a systematic review

**DOI:** 10.3389/or.2024.1427441

**Published:** 2024-11-05

**Authors:** Anna Santos Salas, Nahyeni Bassah, Anna Pujadas Botey, Paula Robson, Julia Beranek, Iqmat Iyiola, Megan Kennedy

**Affiliations:** ^1^ Faculty of Nursing, College of Health Sciences, Third Floor Edmonton, Clinic Health Academy, University of Alberta, Edmonton, AB, Canada; ^2^ Cancer Strategic Clinical Network, Cancer Care Alberta, Alberta Health Services, Foothills Medical Centre, South Tower, Calgary, AB, Canada; ^3^ School of Public Health, Edmonton Clinic Health Academy, University of Alberta, Edmonton, AB, Canada; ^4^ Cancer Strategic Clinical Network, Cancer Care Alberta, Alberta Health Services, Edmonton, AB, Canada; ^5^ Cancer Research and Analytics, Cancer Care Alberta, Alberta Health Services, Edmonton, AB, Canada; ^6^ Geoffrey and Robyn Sperber Health Sciences Library, 1-150M Edmonton Clinic Health Academy, University of Alberta, Edmonton, AB, Canada

**Keywords:** systematic review, health equity, neoplasms, underserved populations, healthcare access, high income countries, universal healthcare

## Abstract

**Background:**

Underserved populations both globally and in Canada face serious cancer inequities that result from systemic economic, environmental, and social conditions. These pose barriers in access to cancer care and lead to suboptimal cancer care experiences and outcomes. Knowledge of effective interventions to improve access to cancer care is needed to inform the design of tailored interventions for these populations.

**Aim:**

To identify interventions and programs to improve access to cancer care for underserved populations in high income countries with universal health coverage (UHC) and the United States (US) throughout the cancer care continuum.

**Methods:**

We conducted a systematic review following the PRISMA standards. We searched Medline, EMBASE, PsycINFO, CINAHL, Scopus, and the Cochrane Library. Inclusion criteria: quantitative and qualitative studies published in English in the last 10 years (2013–2023), describing interventions/programs to improve access to cancer care for underserved populations (18 years and over). We included studies in the US given the body of scholarship on equity in cancer care in that country. Screening, data extraction and analysis were undertaken by two independent reviewers.

**Results:**

Our search yielded 7,549 articles, and 74 met the inclusion criteria. Of these, 56 were conducted in the US, 8 in Australia, 6 in Canada, and 4 in the United Kingdom. Most (90.5%) were quantitative studies and 47.3% were published between 2020–2023. Seven types of interventions were identified: patient navigation, education and counselling, virtual health, service redesign, financial support, improving geographical accessibility and multicomponent interventions. Interventions were mainly designed to mitigate language, distance, financial, lack of knowledge and cultural barriers. Most interventions focused on access to cancer screening, targeted rural populations, racialized groups and people with low socioeconomic status, and were conducted in community-based settings. The majority of interventions or programs significantly improved access to cancer care.

**Conclusion:**

Our systematic review findings suggest that interventions designed to remove specific barriers faced by underserved populations can improve access to cancer care. Few studies came from countries with UHC. Research is required to understand tailored interventions for underserved populations in countries with UHC.

## Introduction

Cancer is among the leading causes of death globally (1). In 2020, there were 19.3 million new cancer cases and 10 million cancer deaths in the world (2). In Canada, cancer is the leading cause of death representing 28.2% of all deaths in 2022 (3). In 2023, 239,100 Canadians were expected to have a cancer diagnosis while 86,700 were expected to die of cancer (4). Two in five Canadians are likely to develop cancer in their lifetime and one in four Canadians are likely to die of cancer (5). Advances in cancer care in Canada have resulted in prolonged survival for some cancers (6). Increases in cancer survival rates are reported for many cancers and across Canadian provinces (4). The 5-year cancer survival index (CSI), an indicator of cancer survival for all cancers, grew 8.4 percentage points from 1992 to 1994 to the 2015–2017 period, nearing 64% (4). Similarly, although the number of cancer deaths has increased, mostly due to population growth and aging, cancer mortality rates have decreased 39% in males and 26% in females since 1988 (4).

Disparities in cancer care represent a significant global challenge (1). In the United States (US), disparities in cancer survival and mortality affecting Blacks and Latino populations are reported (7–9). The lack of universal health coverage (UHC) is associated with inequalities in access to healthcare (10). However, health inequalities in countries with UHC exist (11, 12). In Canada, a country with UHC, progress in cancer care has not been equal for all Canadians. Late cancer diagnoses (13) and lower survival rates are among cancer disparities affecting underserved populations (14, 15). These outcomes are associated with inequalities in access to screening, diagnosis, curative treatment, survivorship care, and palliative care (14, 16, 17). Underserved populations may be overrepresented among those with a late cancer diagnosis (18, 19). For example, incidence rates of lung cancer, the main cause of cancer deaths in Canada, are 1.7 times higher in Canadians living in low-income areas than those living in high-income areas (20). Canadians living with low income also are less likely to receive curative treatment even when diagnosed at an earlier stage (21, 22). The highest rates of lung and colorectal cancer prevalence—the first and second leading causes of cancer deaths in Canada, respectively–were found among people from the lowest income quintiles (6) Cancer disparities affecting rural (13), remote (23), and immigrant populations also exist (13, 24–26). Studies report disparities in breast cancer screening and diagnosis for immigrant women (24–26). Higher rates of mastectomies were reported for women living in rural and remote areas and those with longer travel distances to radiation treatment centres than women living in urban areas and those living closer to radiation treatment centres (14).

Universal health coverage entails the provision of high quality health services, access to high quality health services, and financial risk protection for people who need to use these services (27). The commitment of world leaders to achieve UHC is seen in target 3.8 of the United Nations Sustainable Development Goal #3 “Ensure healthy lives and promote wellbeing for all at all ages” (28). Canada ensures UHC for all Canadians for medically essential hospital, physician, and diagnostic services (29). This entails the provision of care at no charge for these services (29). However, systemic barriers associated with income, race and ethnicity, Indigenous identity, immigration status, geographical location, gender identity, and language, among others, contribute to inequities in access to healthcare (29).

In 2023, the Canadian Cancer Society issued a report identifying 10 underserved communities experiencing systematic disadvantage and barriers in access to cancer information and services as a result of their racial background, gender identity, sexual orientation, geographical location, socioeconomic status, or language, among others (30). In Alberta, the home province of the study authors, low uptake of cancer screening services was identified for low income, rural and remote, gender diverse, and Indigenous populations in this province (31). Studies report successful interventions to support access to cancer care in underserved groups such as comprehensive interprofessional care, intersectoral collaboration, community engagement, empowerment, consultation services, and patient navigation (32–36). However, evidence concerning the types of interventions needed to improve access to cancer care in underserved populations in high income countries with UHC is limited.

To our knowledge, no systematic review of interventions in countries with UHC has been conducted. Increasing our understanding of these interventions and their impact could help inform strategies to improve cancer equity and outcomes in underserved populations in Alberta and Canada (37). In collaboration with Cancer Care Alberta stakeholders (APB, PR), we designed a systematic review of interventions to improve access to cancer care throughout the cancer care continuum in underserved populations in high income countries with UHC, with a view to inform the design of tailored interventions in our province. We included studies from the US considering scholarship in the area in this country and policy changes resulting in increased access to healthcare for underserved communities (10).

## Methods

### Population, interventions, comparison group and outcome

The population for this study was adult patients (18 years and older) diagnosed with cancer at any stage from underserved populations in high income countries with UHC and the US. Studies with Indigenous peoples were excluded.

The interventions of interest for this review were any intervention with the goal to increase access to cancer care along the cancer care continuum (38, 39). We included formal evaluations of programs that had the same goal. Interventions that addressed access to healthcare dimensions (40), such as availability and accommodation, approachability, accessibility, acceptability, and affordability of services were included.

### Data sources and search strategy

Our review is reported in adherence to the PRISMA statement, the PRISMA for Searching (PRISMA-S) extension (41). Methodological guidance was taken from the Cochrane Handbook for Systematic Reviews of Interventions (42).

In order to identify all relevant published studies, a comprehensive, systematic search was conducted by a health sciences librarian (MK) familiar with systematic review methodology. Searches were conducted using the following bibliographic databases on 10 May 2023: Medline, EMBASE, and PsycINFO via OVID; Cumulative Index to Nursing and Allied Health Literature (CINAHL) via EBSCOhost; Scopus; and the Cochrane Library via Wiley. All databases were searched from inception to present. The search strategy was derived from three main concepts: 1) Vulnerable populations including people living in rural communities, people with intellectual disabilities, people with physical disabilities or mobility problems, people with lower socioeconomic status, and people from racially marginalized groups; 2) Cancer care including treatment, management, and surgical care; 3) Access to healthcare services or health services accessibility. The search strategies for each database were constructed using a combination of natural language keywords and subject headings, such as MeSH, wherever they were available. Results were focused geographically on Canada, Australia, New Zealand, the United Kingdom, Scandinavian nations, and the United States. Limits of English language and publication date 2013–2023 were applied. We limited the search to the last 10 years to ensure review feasibility. Randomized controlled trials, controlled trials, before-and-after studies and interrupted time series (with or without control) and observational, qualitative, or mixed methods were included. Additionally, publication types of case reports, comments, letters, editorials, conference materials, and news items were removed from the results. See Supplementary Appendix 1 for full search strategies for each database.

Results were exported in complete batches from the databases on 10 May 2023. The synthesis review management software, Covidence^©^, was used to remove duplicate records and manage the title/abstract and full-text screening phases of the review. The reference list of all included articles was searched for additional studies. We also conducted a Scopus citation chaining to identify other potentially eligible articles and a focused grey literature search on PubMed and Google to identify additional Canadian studies.

### Study selection process

The review team was trained by the senior team members (NB, AS) to ensure consistency with review processes. First, titles and abstracts were screened by two independent reviewers. Secondly, full-texts of potentially eligible studies were assessed for eligibility by two independent reviewers. Disagreements were resolved through discussion and by a third senior team member (AS, NB).

### Quality assessment and risk of bias

Four reviewers (NB, JB, IqI, HJ) were involved in quality appraisal of included articles, with two independent reviewers assessing the methodological quality of each included study. We employed the Quality Assessment Tool for Quantitative Studies (43), and the Critical Appraisal Skills Programme (CASP) Qualitative Studies Checklist (44).

### Data synthesis and analysis

Data were extracted using a structured form developed based on review objectives. Categories included authors and year of publication, country, aim, design, cancer care setting, population characteristics, type of intervention, intervention characteristics, and outcomes. We undertook thematic analysis (45) of the findings and produced a narrative synthesis of the themes. Interventions and programs were grouped according to shared characteristics and were organized under a primary theme. We undertook a separate analysis of Canadian studies.

## Results

### Study characteristics

**FIGURE 1:**
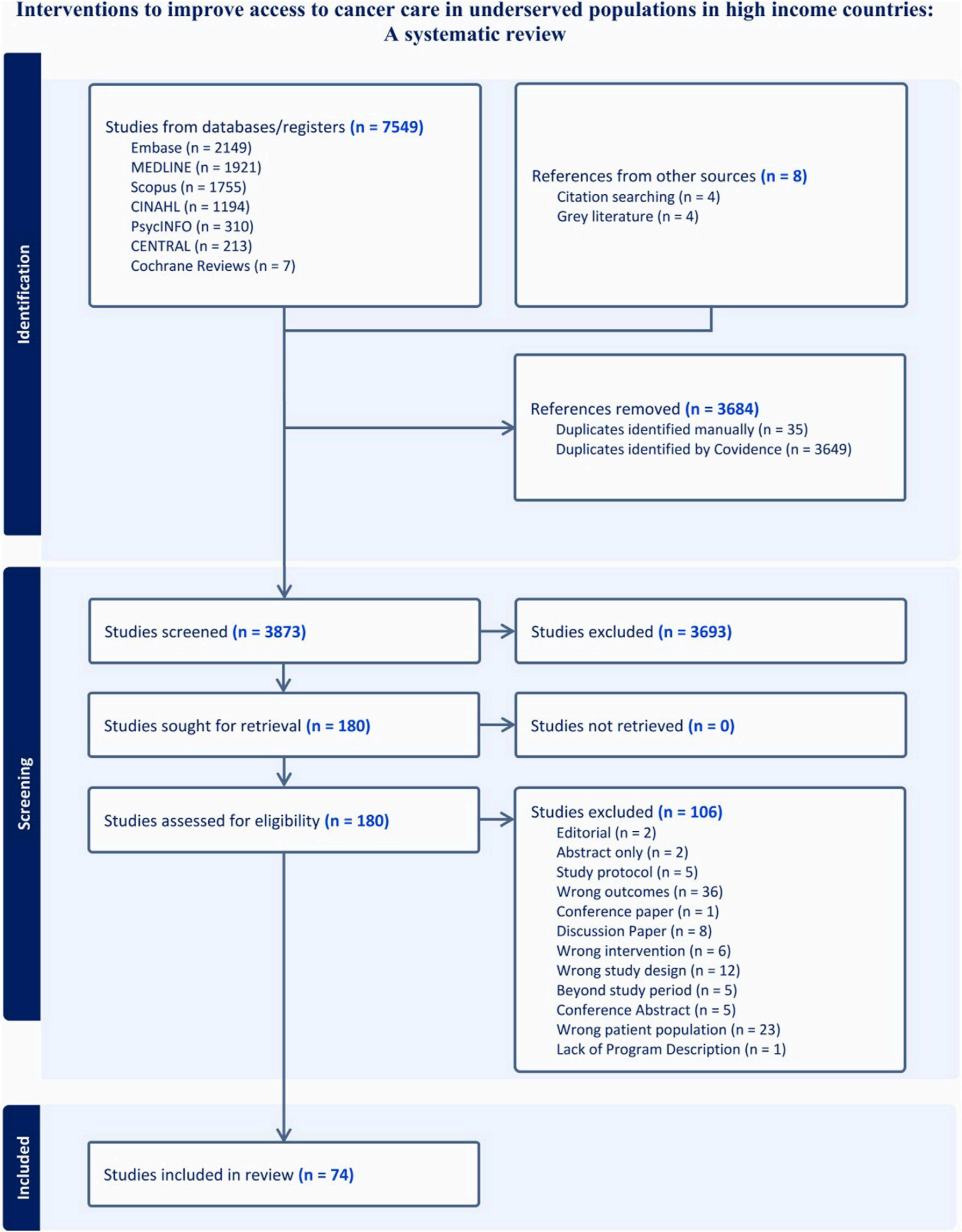
PRISMA diagram.

We obtained 7,549 articles from database searches and 8 from citation chaining and grey literature. A total of 3,684 duplicates were removed. We screened the titles and abstracts of 3,873 articles and 3,693 did not meet our inclusion criteria. A total of 180 full texts were screened and 74 met the inclusion criteria (Figure 1). Of the 74 studies, 56 were conducted in the US, 8 in Australia, 6 in Canada, and 4 in the United Kingdom. They were published between 2013 and 2023. There was an increase in studies describing access to cancer care interventions for underserved populations in the literature, with 47% published in the last 3 years (2020–2023) (Table 1).

**TABLE 1:** Sociodemographic characteristics of study populations.

Characteristics	N (%)
Publication dates	
• 2013–2019	39 (52.7)
• 2020–2023	35 (47.3)
Country	
• United States of America	56 (76.0)
• Australia	8 (11.0)
• Canada	6 (8.0)
• United Kingdom	4 (5.0)
Setting	
• Community-based	55 (74.3)
• Cancer centres and hospitals	15 (20.2)
• Virtually with no specific setting identified	2 (2.7)
• Hospice	1 (1.4)
• Correctional setting	1 (1.4)
Underserved group	
• Low socioeconomic status	26 (35)
• Rural	26 (35)
• Racialized (African American/Black, Latino/as, Vietnamese, Asian including Chinese, Korean)	12 (16)
• Others (Underserved women, underserved men, veteran, uninsured, incarcerated people)	10 (14)
Type of cancer	
• Mixed diagnoses	28 (37.9)
• Breast	19 (25.6)
• Colorectal	12 (16.2)
• Lung	5 (6.8)
• Cervical	4 (5.4)
• Others	6 (8.1)
Study design	
• Observational: retrospective and prospective	39 (58.2)
• Quasi-experimental controlled or pretest and/or post-test single group	15 (22.4)
• Randomized controlled trials (RCTs)	13 (19.4)
• Qualitative	4 (5.4)
• Mixed methods	3 (4.1)
Type of intervention or program	
• Multicomponent	22 (29.7)
• Virtual health	14 (18.9)
• Navigation	12 (16.2)
• Service redesign	10 (13.5)
• Education and counselling	7 (9.5)
• Financial support	5 (6.8)
• Reducing geographical distance	4 (5.4)
Cancer care continuum	
• Screening	50 (67.6)
• Cancer treatments	15 (20.3)
• Genetic counselling	3 (4)
• Psychosocial and supportive care	3 (4)
• Palliative care	2 (2.7)
• Survivorship care	1 (1.4)
Key outcomes	
• Access to screening services	24 (32.4)
• Access to cancer treatment	25 (33.8)
• Patient satisfaction	13 (17.5)
• Increased knowledge	8 (10.8)
• Access to psychosocial, palliative and supportive care	3 (4)
• Access to genetic counselling	3 (4)

Sixty-seven studies (90.5%) were quantitative, 4 (5.4%) were qualitative (46–49) and 3 (4.1%) were mixed methods (50–52). Among the quantitative studies, 13 (19.4%) were randomized controlled trials (RCTs) (53–65), 15 (22.4%) were quasi-experimental controlled or pretest and/or post-test single group studies (66–80), and 39 (58.2%) were observational including natural experiments, and retrospective and/or prospective analyses of service data.

Sample sizes were above 100 participants for most studies, ranging from 3 to 190,284 participants, with the largest samples registered in retrospective studies. In most studies, participants were 50 years and older (Table 2).

**TABLE 2:** Summary of included studies.

Author/Date	Country	Aim/objective	Settings	Cancer type	Sample characteristics
Arnold et al. 2022	Australia	To provide gynaecological oncology consultations	Community-based Gynecologic oncology clinic	Gynecologic	Sample size: 53 Age: 21–84 years Rural and remote women
Azizoddin et al. 2020	United States	To improve screening for cancer‐related distress and access to supportive care	Oncology Clinic	Mixed	Sample size: 253 Age: 33–99 Veterans who were mostly African American and males of low SES
Baker et al. 2014	United States	To determine whether a multifaceted intervention increases adherence to annual FOBT for CRC screening compared with usual care	Community health centres	Colorectal	Sample size: 450 Age: 51–75 years Latino; low income and uninsured
Balata et al. 2019	United Kingdom	To ascertain the views of participants of a mobile CT scan	Convenient community retail locations in deprived areas	Lung	Sample size: 938 Age: 55–74 years Low-income
Battaglia et al. 2019	United States	To address socio-legal barriers for patients with cancer	Community health centres and outpatient practices	Mixed	Sample size: 306 Age: >18 years Low SES, racialized
Centra et al. 2023	United States	To improve access to high-quality CRC screenings and reduce barriers to test completion	Federally Qualified Health Centre	Colorectal	Sample size: 20 Age: 50–75 years Low SES: unhoused, marginally housed, or recent refugees
Champion et al. 2023	United States	To assess the comparative effectiveness of (1) a mailed, tailored digital video disc (DVD) intervention; (2) a DVD intervention plus telephone patient navigation (DVD/PN); and (3) usual care with simultaneously increased adherence to any breast, cervical, and CRC screening	Rural community	Mixed	Sample size: 963 Age: 50–74 years Women in rural communities
Charlton et al. 2014	United States	To determine if FIT mailed to asymptomatic, average-risk patients overdue for screening resulted in higher screening rates versus mailing educational materials alone or no intervention	Veterans administration Medical centre	Mixed	Sample size: 1,999 Age: 51–64 years Rural
Curtis et al. 2018	United States	To analyze a satellite chemotherapy infusion centre	Community- and hospital- based	Mixed	Sample size: 3 Age: Not reported Rural
Davis, et al. 2020	United States	To evaluate the effectiveness of two approaches to a health literacy intervention to improve annual CRC screening: automated telephone call or personal call	Rural community health clinics	Colorectal	Sample size: 568 Age: ≥50 years Rural
Denizard-Thompson et al. 2020	United States	To determine the impact of a Mobile Patient Technology for Health- CRC on receipt of CRC screening within 24 weeks	Community-based primary care practices	Colorectal	Sample size: 450 Age: 50–74 years Low SES: Rural, African American, Low income, Low literacy
Drake et al. 2022	United States	To develop outreach and education, quality improvement and research, and training strategies to lessen the burden of cancer disparities	Cancer centre and community organizations	Breast	Sample size: 8,292 Age: ≥21 years Socioeconomically deprived, racial minority women
Dumont et al. 2021	United States	To implement and evaluate a population health approach to preventive care in correctional setting	Correctional facility	Colorectal	Sample size: > 2,700 Age: 50 years or ≥45 years for Blacks Racialized, incarcerated People
Eberth et al. 2018	United States	To provide access to colonoscopy screening at no cost	Medical clinics, and hospitals	Colorectal	Sample size: 1,854 Age: 50–64 years Uninsured, or live at or below 150% poverty line
Falk et al. 2023	United States	To educate women about the need for breast and cervical cancer screenings and to offer navigation services to reduce financial and other barriers to Pap tests and mammograms	Community-based	Mixed	Sample size: 1,181 Age: 18–39 years Rural
Fang et al. 2017	United States	To increase awareness of cervical cancer risk and address both individual-level beliefs and healthcare access barriers	Korean churches	Cervical	Sample size: 977 Age: ≥21 years Korean women
Fennell et al. 2017	Australia	To describe a consumer-led development of a website that provides rural-specific information on psychosocial care and examine its acceptability to users	Virtual, with no specific setting	Mixed	Sample size: 32,389 Age: Not reported Rural
Foley et al. 2023	Australia	To shift care from the tertiary cancer centre to local services for rural people with head and neck surgery	Rural clinics	Head and neck	Sample size: 5 Age: Not reported Rural
Freund et al. 2013	United States	To investigate the efficacy of patient navigation in reducing delays in resolving abnormal cancer screening tests and initiating cancer treatment	Community-based	Mixed	Sample size: 10,521 Age: ≥18 years Low-income, uninsured or publicly insured, and racial minority
Galiatsatos et al. 2021	United States	To examine the utility of a tobacco treatment clinic that provides lung cancer screening, for minority populations	Tobacco treatment clinic at a medical centre	Lung	Sample size: 92 Age: ≥50 years Racialized, low SES
Gunness et al. 2023	United States	To analyze the impact of single breast reconstruction service and inform consumers and providers of the importance of a breast reconstruction unit embedded in a rural health network	Breast reconstruction service in a rural health network	Breast	Sample size: 64 Age: Not reported Rural women
Hall et al. 2019	Australia	To trial and evaluate a compression garment service that provides care for patients with lymphoedema closer to their homes	Rural and remote health services	Skin	Sample size: 69 Age: ≥16 years Rural and remote
Hendren et al. 2014	United States	To assess an intervention to increase cancer screening among patients	An inner-city large family practice	Mixed	Sample size: 366 Age: 40–74 years Low-income and uninsured patients
Hitt et al. 2016	United States	To review the accessibility to care and its impact of a tele-colposcopy	Hospital	Cervical	Sample size: 940 Age: 17–62 years Rural women
Holle et al. 2020	United States	To assess the use of community pharmacist as the primary healthcare team member to facilitate CRC risk counseling and screening	Pharmacies in hospitals, large physician practice, or within the community	Colorectal	Sample size: 60 Age: ≥50 years or African Americans aged ≥45 years Low SES, racialized
Hoskins et al. 2018	United States	To test the feasibility of implementing the US Preventive Services Task Force’s recommendation for universal cancer genetic risk assessment by primary care physicians	Community clinics	Mixed	Sample size: 112 Age: 25–69 years Underserved African American women
Humer et al. 2017	Canada	To use telemedicine, through Virtual Thoracic Surgical Clinics, to provide service to remote patients	Community-based	Thoracic	Sample size: 15,073 Age: Not reported Rural and remote
Johnson et al. 2021	United States	To evaluate differences in time from breast cancer diagnosis to treatment for women enrolled in a Women’s Health Check program	Cancer Data Registry	Breast	Sample size: 231 Age: 50–64 years Low-income, uninsured and underinsured women
Khalil et al. 2020	United States	To determine the rates of breast cancer screening at a student-run free clinic and compare them to national averages	Academic health centre	Breast	Sample size: 194 Age: ≥40 years Uninsured women
Kim et al. 2020	United States	To improve CRC screening uptake	Community-based	Colorectal	Sample size: 3,610 Age: Based on US CRC eligibility guidelines Diverse underserved urban patient population
Kiser et al. 2020	United States	To have 75% of the women who sought care at the study clinic during the 60-day project period receive Pap test eligibility screening	Federally qualified health centre	Cervical	Sample size: 6,900 Age: 21–65 years Uninsured and underserved women
Lane et al. 2015	United States	To provide breast cancer screening and education	Community-based	Breast	Sample size: 2,394 Age: ≥50 years Rural
Lara et al 2018	United States	To assess changes in CRC screening uptake and the cost-effectiveness of implementing multiple evidence-based interventions	Hospital-based	Colorectal	Sample size: 8,277 Age: Not reported Underserved adults
Lee-Lin et al. 2013	United States	To assess the feasibility and acceptability of a targeted educational intervention to increase mammography screening	Chinese community partner agencies	Breast	Sample size: 44 Age: ≥40 years Chinese American immigrant women
Le et al. 2022	United States	To develop and implement a community-based lung cancer screening program, including telephone-based navigation and tobacco cessation counseling support	Communities in rural and medically underserved counties	Lung	Sample size: 488 Age: 55–77 years Low-income, uninsured or underinsured patients
Limaye et al. 2022	United Kingdom	To ensure >95% of male patients over the age of 65 or those at high risk of developing prostate cancer have had a prostate-specific antigen test; or have been referred if abnormal	Community-based GP Practice	Prostate	Sample size: 220 Age: ≥65 years Males
Li et al. 2019	United States	To increase the breast cancer screening rate of Hispanic women	Hospital District	Breast	Sample size: 30 Age: ≥30 years
Lofters et al. 2017	Canada	To increase awareness of cancer susceptibility and the benefits of screening for breast and cervical cancer	Black-focused Community Health Centre	Mixed	Sample size: 30 Age: 40–69 years Black women
Luckett et al. 2015	United States	To assess the effect of Patient Navigator Program on no-show rates at an academic referral colposcopy Clinic	Specialty clinic within the Gynecologic Oncology Department at Brigham and Women’s Hospital	Cervical	Sample size: 4,199 Age: 33 years average Women: Low SES, history of abuse racialized
Manning et al. 2023	United States	To assess the effects of a racially-targeted messaging intervention to increase uptake of at-home FIT kits for CRC screening	Community-based	Colorectal	Sample size: 1,157 Age: 45–80 years African American
Mayfield-Johnson et al. 2016	United States	To increase the breast cancer screening rate for African American women	Community-based	Breast	Sample size: 500 Age: ≥40–64 years African American women
Menon et al. 2020	United States	To test the effectiveness of a community-to-clinic navigator intervention to guide individuals into primary care clinics to complete CRC screening	Community-based	Colorectal	Sample size: 419 Age: ≥50 years Multicultural and underinsured
Mette et al. 2016	United States	To provide cancer genetic risk assessment and counseling through telemedicine to the remote population	Community-based	Mixed	Sample size: 119 Age: ≥20 years Low SES: rural, uninsured or underinsured
Molokwu et al. 2023	United States	To determine the effectiveness of a multilevel, multicomponent community-based breast cancer screening intervention	Community-based: Two clinics and 59 communities	Breast	Sample size: 600 Age: 50–75 years Hispanic Women
Niranjan et al. 2023	United States	To assess lung cancer screening knowledge before and after receiving education delivered by community health advisors	Community-based	Lung	Sample size: 100 Age: 50–80 years Rural, poor, unemployed
Nnorom et al. 2021	Canada	To improve cancer screening for Blacks and immigrants	Black-focused Community Health Centre	Mixed	Sample size: 708 Age: Not reported Black and immigrant population
Offman et al. 2014	United Kingdom	To remind underserved women to attend breast cancer screening via telephone	Community-based	Breast	Sample size: 10,928 Age: 50–70 years High deprivation and ethnic diverse women
Patel et al. 2023	United States	To decrease the average number of treatment day delays during the first six cycles of oral cancer	Medical centre	Mixed	Sample size: 53 Age: 29–88 years Low SES and racialized women
Peppercorn et al. 2017	United States	To evaluate the elimination of cost sharing as a natural experiment	Rural clinics	Breast	Sample size: 45,738 Age: 40–64 years Rural women
Percac-Lima et al. 2014	United States	To evaluate the impact of a CRC screening patient navigation program on improving equity in CRC screening	Primary care practice	Colorectal	Size: 3,115 Age: 61.5 years average Low-income, Latino and immigrant population
Pye et al. 2023	Australia	To explore experiences of rural cancer patients who were receiving treatments by remote video-assisted chemotherapy	Community-based	Mixed	Sample size: 7 Age: 39–71 years Rural
Rajan et al. 2015	United States	To address socioeconomic disparities in breast and cervical cancer screening and survival	Cancer Registry	Mixed	Sample Size: 190,284 Age: 40–64 years Underserved women
Ramirez et al. 2014	United States	To apply a patient navigation model to women with an abnormal mammogram to determine its effectiveness in reducing time from abnormal breast examination findings to definitive diagnosis, and to evaluate its effect on time from definitive diagnosis to initiation of treatment	Community-based	Breast	Sample size: 480 Age: ≥18 years Latino women
Richman et al. 2020	United States	To provide culturally tailored breast cancer education and navigation to age-appropriate screening services	Community-based	Breast	Sample size: 735 Age: 20–84 years Uninsured and underinsured Black and Latina women
Sabesan et al. 2014	Australia	To provide cancer care closer to home in a timely and equitable manner for rural patients	Cancer centre	Mixed	Sample size: 60 Age: Not reported Rural
Sabesan et al. 2018	Australia	To overcome barriers and establish equitable access to safe and quality chemotherapy services locally in rural towns	Hospital and rural satellite sites	Mixed	Sample size: 62 Age: Not reported Rural
Sanchez-Birkhead et al. 2016	United States	To address the needs of Hispanic women faced with a cancer diagnosis or cancer survivorship issues	Community-based	Breast	Sample size: >8,000 Age: 30–70 years Hispanic women
Schroeder et al. 2021	United States	To assess oral cancer knowledge and provide oral cancer screening and education	Community-based	Oral	Sample size: 236 Age: ≥18 years Rural
Solomons et al. 2018	United States	To examine knowledge and emotional outcomes and attitudes/beliefs regarding cancer tele-genetic service	Medical Centre Cancer Risk and Prevention Clinic	Mixed	Sample size: 174 Age: Not reported Rural
Swayze et al. 2021	United States	To provide access to gynecologic oncology care to ovarian cancer patients in small cities and rural communities	Gynecologic oncology office	Ovarian	Sample size: 381 Age: ≥18 years Rural
Thai et al. 2022	United States	To shift the attention from increasing initial mammography rates to improving appropriate follow-up after an abnormal mammogram	Community-based	Breast	Sample Size: 96 Age: 62 years average Vietnamese-American Women
Thota et al. 2020	United States	To care for patients in rural communities to improve access to healthcare, decrease financial burdens, and save time	Rural communities	Mixed	Sample size: 119 Age: Not reported Rural
Tracy et al. 2013	United States	To examine how visiting consultant clinics in rural communities affected estimated average travel times for rural residents	Community clinics	Mixed	Sample size: 18 Age: Not reported Rural
Tsai et al. 2014	United States	To improve access to primary healthcare for various populations	Nurse-managed health centres	Breast	Size: 577 Age: ≥18 years Racialized and low SES women
Tsapatsaris and Reichman 2021	United States	To evaluate whether, with access to free screening services, uninsured minority women are able to successfully manage existing barriers to breast cancer screening	Community-based	Breast	Sample size: 3,745 Age: Not reported Low-income uninsured minority women
van den Bruele et al. 2022	United States	To provide a free screening service to women in need	Community-based	Breast	Sample size: 32,350 Age: 40–79 years Underserved women
Vilchis et al. 2019	United States	To assess the detailed cancer navigation needs of patients and their families in an underserved target area	Medical centre or cancer treatment centre	Mixed	Sample Size: 128 Age: ≥18 years Rural
Wagoner et al. 2023	Canada	To support exercise-oncology implementation in rural and remote communities across Canada	Virtual, with no specific setting	Mixed	Sample size: 290 Age: ≥18 years Rural
Wakefield et al. 2023	United Kingdom	To describe the development of a new innovative long-term palliative care unit	Hospice	Mixed	Sample size: 199 Age: ≥24 years Socio-economically deprived
Watanabe et al. 2013	Canada	To assess the feasibility of using videoconferencing to provide specialist multidisciplinary palliative care and palliative radiotherapy consultation to cancer patients in rural areas	Cancer centre	Mixed	Sample size: 44 Age: 20–88 years Rural
Watson et al. 2016	Canada	To improve rural Albertans’ access to navigation supports	Ambulatory care-community	Mixed	Sample size: 81 Age: Not reported Rural
Williams MS et al. 2022	United States	To provide uninsured, underserved women with access to a free mammogram, a Pap test and pelvic exam, and/or an oral cancer exam	Medical Centre Cancer Centre and Research Institute	Mixed	Sample size: 103 Age: 21–69years Low SES women
Williams MA et al.2022	United States	To help guide patients with the goal of improving care coordination, psychosocial care, patient education, and healthcare usage	Comprehensive cancer Centre	Mixed	Sample size: 54 Age: Not reported Rural, low SES, racialized
Wilson-Anderson et al. 2013	United States	To provide breast health education	Community-based faith-based groups and women’s social organizations	Breast	Sample size: 130 Age: 11–73 Mostly African American

CRC, colorectal cancer; FIT, fecal immunochemical test; FOBT, fecal occult blood testing; GP, general practitioner; SES, socioeconomic status.

Most studies were community-based (74.3%) while others took place in cancer centres and hospitals (47, 71, 75, 81–92), hospice (93), correctional service (70), and with no specific setting identified (51, 67) (Tables 1, 2).

Access to cancer care was the primary outcome in most studies, but 8.1% of included studies reported access as a secondary outcome (73, 74, 76, 82, 94, 95) Measures of access included access to cancer screening (screening rates, no show rates, appointment rates); access to cancer treatment (time from diagnosis to treatment initiation, adherence to treatment and follow ups, proportion of patients accessing cancer treatment or supportive care); out-of-pocket and time savings; and travel distance to cancer care services. Knowledge and patient satisfaction were reported as secondary outcomes. Supplementary Table 1 provides an overview of study outcomes by intervention category and individual studies.

### Characteristics of underserved populations

The most common underserved groups targeted by included studies were rural populations and people experiencing socioeconomic deprivation. Other studies were focused on Blacks and African Americans (65, 73, 95–97), Latino/Hispanics (52, 69, 79, 98), Asian women including Chinese (72) Vietnamese (99), and Korean women (100), underserved women (82, 101, 102), underserved men (103), veterans (54, 81), incarcerated people (70) and uninsured patients (92, 104, 105) (Tables 1, 3). In many cases, study populations had mixed characteristics such as low income and racialized background.

**TABLE 3:** Quality appraisal of quantitative studies^a^.

Author and date	Selection bias	Study design	Confounders	Blinding	Data collection methods	Withdrawals and drop-outs	Global rating
Arnold et al. 2022	Strong	Moderate	Weak	Moderate	Strong	Moderate	Moderate
Azizoddin et al. 2020	Moderate	Moderate	Weak	Weak	Strong	Weak	Weak
Baker et al. 2014	Strong	Strong	Strong	Moderate	Moderate	Strong	Strong
Balata et al. 2019	Moderate	Moderate	Strong	Moderate	Moderate	Strong	Strong
Battaglia et al. 2019	Moderate	Moderate	Strong	Moderate	Moderate	Strong	Strong
Centra et al. 2023	Moderate	Moderate	Strong	Moderate	Strong	Weak	Moderate
Champion et al. 2023	Strong	Strong	Strong	Moderate	Moderate	Strong	Strong
Charlton et al. 2014	Strong	Strong	Strong	Strong	Moderate	Weak	Moderate
Davis et al. 2020	Strong	Strong	Strong	Moderate	Moderate	Moderate	Strong
Denizard-Thompson et al. 2020	Moderate	Strong	Strong	Moderate	Strong	Moderate	Strong
Drake et al. 2022	Strong	Moderate	Strong	Moderate	Strong	Moderate	Strong
Dumont et al. 2021	Moderate	Moderate	Weak	Moderate	Weak	Moderate	Weak
Eberth et al. 2018	Strong	Moderate	Strong	Moderate	Strong	Weak	Moderate
Falk et al. 2023	Moderate	Moderate	Strong	Moderate	Weak	Strong	Moderate
Fang et al. 2017	Strong	Strong	Strong	Moderate	Strong	Strong	Strong
Fennell et al. 2017	Moderate	Moderate	Weak	Moderate	Moderate	Moderate	Moderate
Freund et al. 2013	Strong	Strong	Strong	Strong	Moderate	Strong	Strong
Galiatsatos et al. 2021	Moderate	Moderate	Strong	Moderate	Weak	Strong	Moderate
Gunness et al. 2023	Moderate	Moderate	Weak	Moderate	Strong	Moderate	Moderate
Hall et al. 2019	Moderate	Weak	Weak	Moderate	Weak	Weak	Weak
Hendren et al. 2014	Moderate	Strong	Strong	Strong	Strong	Weak	Moderate
Hitt et al. 2016	Weak	Moderate	Weak	Strong	Weak	Weak	Weak
Holle et al. 2020	Weak	Moderate	Strong	Moderate	Weak	Strong	Weak
Hoskins et al. 2018	Moderate	Moderate	Moderate	Moderate	Weak	Strong	Moderate
Humer et al. 2017	Moderate	Weak	Weak	Moderate	Weak	Weak	Weak
Johnson et al. 2021	Moderate	Moderate	Strong	Moderate	Strong	Moderate	Strong
Khalil et al. 2020	Moderate	Moderate	Strong	Moderate	Weak	Weak	Weak
Kim et al. 2020	Moderate	Moderate	Strong	Moderate	Weak	Weak	Weak
Kiser et al. 2020	Moderate	Moderate	Weak	Moderate	Moderate	Strong	Moderate
Lane et al. 2015	Moderate	Moderate	Weak	Moderate	Weak	Weak	Weak
Lara et al. 2018	Weak	Moderate	Weak	Moderate	Weak	Weak	Weak
Lee-Lin et al. 2013	Moderate	Moderate	Strong	Strong	Strong	Strong	Strong
Le et al. 2022	Moderate	Moderate	Strong	Moderate	Weak	Strong	Moderate
Limaye et al. 2022	Moderate	Moderate	Weak	Moderate	Weak	Strong	Weak
Li et al. 2019	Weak	Moderate	Strong	Moderate	Moderate	Weak	Weak
Lofters et al. 2017	Moderate	Moderate	Weak	Moderate	Weak	Strong	Weak
Luckett et al. 2015	Strong	Moderate	Strong	Strong	Moderate	Moderate	Strong
Manning et al. 2023	Strong	Strong	Strong	Moderate	Strong	Moderate	Strong
Mayfield-Johnson et al. 2016	Weak	Moderate	Weak	Moderate	Weak	Strong	Weak
Menon et al. 2020	Weak	Moderate	Moderate	Moderate	Moderate	Moderate	Moderate
Mette et al. 2016	Weak	Moderate	Moderate	Moderate	Moderate	Weak	Weak
Molokwu et al. 2023	Moderate	Moderate	Strong	Moderate	Strong	Strong	Strong
Niranjan et al. 2023	Moderate	Moderate	Strong	Moderate	Strong	Strong	Strong
Nnorom et al. 2021	Moderate	Moderate	Weak	Moderate	Strong	Weak	Weak
Offman et al. 2014	Weak	Moderate	Strong	Moderate	Weak	Weak	Weak
Patel et al. 2023	Weak	Moderate	Strong	Moderate	Weak	Weak	Weak
Peppercorn et al. 2017	Moderate	Strong	Weak	Moderate	Moderate	Weak	Weak
Percac-Lima et al. 2014	Moderate	Strong	Weak	Moderate	Strong	Weak	Weak
Rajan et al. 2015	Strong	Moderate	Strong	Moderate	Weak	Strong	Moderate
Ramirez et al. 2014	Moderate	Strong	Strong	Moderate	Moderate	Strong	Strong
Richman et al. 2020	Moderate	Moderate	Strong	Moderate	Weak	Strong	Moderate
Sabesan et al. 2014	Moderate	Weak	Moderate	Moderate	Weak	Weak	Weak
Sabesan et al. 2018	Moderate	Weak	Strong	Moderate	Weak	Strong	Weak
Sanchez-Birkhead et al. 2016	Strong	Moderate	Strong	Moderate	Strong	Strong	Strong
Schroeder et al. 2021	Strong	Moderate	Strong	Moderate	Strong	Strong	Strong
Solomons et al. 2018	Moderate	Moderate	Strong	Moderate	Moderate	Moderate	Strong
Swayze et al. 2021	Strong	Moderate	Strong	Moderate	Moderate	Strong	Strong
Thai et al. 2022	Moderate	Moderate	Strong	Moderate	Strong	Strong	Strong
Thota et al. 2020	Weak	Moderate	Weak	Moderate	Weak	Weak	Weak
Tracy et al. 2013	Moderate	Moderate	Weak	Moderate	Strong	Weak	Weak
Tsai et al. 2014	Moderate	Moderate	Moderate	Moderate	Moderate	Strong	Strong
Tsapatsaris and Reichman 2021	Weak	Moderate	Weak	Moderate	Weak	Moderate	Weak
van den Bruele et al. 2022	Strong	Moderate	Strong	Moderate	Strong	Strong	Strong
Vilchis et al. 2019	Strong	Moderate	Strong	Moderate	Strong	Strong	Strong
Wagoner et al. 2023	Moderate	Weak	Weak	Moderate	Weak	Strong	Weak
Wakefield et al. 2023	Moderate	Weak	Weak	Moderate	Weak	Strong	Weak
Watanabe et al. 2013	Moderate	Moderate	Weak	Moderate	Moderate	Moderate	Moderate
Williams MS et al. 2022	Moderate	Weak	Weak	Moderate	Weak	Weak	Weak
Williams MA et al. 2022	Moderate	Moderate	Weak	Moderate	Moderate	Weak	Weak
Wilson-Anderson et al. 2013	Strong	Moderate	Weak	Weak	Moderate	Weak	Weak

^a^ Quality appraisal tool: Effective Public Healthcare Panacea Project. Quality Assessment Tool for Quantitative Studies. 2023; https://www.ephpp.ca/quality-assessment-tool-for-quantitative-studies/.

### Types of cancers

The majority of included studies either included people with any type of cancer (mixed) or were focused on breast (69, 72, 75, 79, 80, 87, 95, 97, 98, 99, 101, 104, 106–111), cervical (86, 88, 100, 102) , and colorectal cancers (53–55, 58, 60, 61, 65, 70, 76, 77, 78, 105), or a combination of these three cancers (62, 64, 73, 82, 112). The rest of the studies were focused on lung (56, 68, 74, 113, 114) thoracic (115), ovarian (116), skin (59), oral (71), head and neck (48), and prostate cancers (103) (Tables 1, 2).

### Targeted stage across the cancer care continuum

Cancer screening was the stage of focus for most studies, with a few focusing on genetic counselling (66, 94, 96), psychosocial and supportive care (51, 75, 81), survivorship care (98), and palliative care (85, 93). There were 15 studies that aimed to improve access to cancer treatments specifically with respect to: treatment initiation (57, 89), post acute care (48), chemotherapy and medical oncology (46, 47, 84, 117), thoracic surgery (115), gynaecologic oncology (50, 116), exercise oncology (67), treatment adherence and toxicity management (99), lymphedema management (59), breast reconstruction (108), and oral cancer care (71) Supplementary Table 1.

### Quality of included studies

Among the quantitative studies, 24 were appraised to have an overall strong quality, 16 were of moderate quality and 27 of weak quality (Table 3). The study designs used in most studies were of strong to moderate quality, and included participants who were representative of the target populations. Some methodological limitations found were the poor reporting of numbers and reason for dropouts (77, 105, 108, 118), lack of information about reliability and validity of data collection tools, (71, 104, 113) and poor reporting about control of confounders (67, 82, 93). Regarding the qualitative studies, two studies met all the quality criteria (46, 48, 49) one met 8/9 criteria, while one did not meet the majority of the quality criteria (47) (Table 4).

**TABLE 4:** Quality appraisal of qualitative studies^a^.

Measure	Curtis et al.	Foley et al.	Pye et al.	Watson et al.
Clear statement of research aims	Y	Y	Y	Y
Appropriateness of qualitative methodology	Y	Y	Y	Y
Appropriateness of research design	N	Y	Y	Y
Appropriateness of recruitment strategy	N	Y	Y	Y
Appropriateness of data collection strategy	N	Y	Y	Y
Consideration of the relationship between researcher and participants	N	Y	Y	Can’t tell
Ethical considerations addressed	N	Y	Y	Y
Sufficiently rigorous data analysis	N	Y	Y	Y
Clear statement of findings	Y	Y	Y	Y

^a^Quality appraisal tool: Critical Appraisal Skills Programme. CASP Qualitative Studies Checklist. 2018; https://casp-uk.net/images/checklist/documents/CASP-Qualitative-Studies-Checklist/CASP-Qualitative-Checklist-2018_fillable_form.pdf.

### Interventions to improve access to cancer care

We found seven main types of interventions that were used to mitigate barriers in access to cancer care experienced by underserved populations. These included lack of knowledge and health literacy, health system navigation issues, financial constraints, language and cultural barriers, and distance to cancer services. These tailored interventions included patient navigation, education and counselling, virtual health, service-redesign, financial support, improving geographical accessibility and multicomponent interventions. These are described below.

### Core features of interventions

Salient characteristics of interventions included: 1) Most interventions/programs used a combination of languages and offered translation services to enhance communication and understanding. The most common languages used were English, Spanish, and French; 2) Participant recruitment was via community organizations, churches, local bulletin boards, community centres, health fairs, community events, food banks and social medial platforms, using flyers and leaflets which sometimes featured racialized groups (60, 72, 73, 91, 119); 3) There was a predominant use of the community health approach to program development and delivery with significant involvement of community health workers and peers for education and navigation (80, 98); 4) Several interventions and programs included reminder phone calls, mailed letters, and electronic medical record alerts, setting reminders to both patients and clinicians, invitations to patients overdue for screening and/or those who did not attend their appointments as well as to request fecal immunochemical test (FIT) patient samples (61, 64, 65, 78–80, 98, 100, 103, 119); 5) Most programs were delivered to participants at no charge or low cost (79, 80, 98, 100, 114); 6) Some studies included all participants who met the inclusion criteria irrespective of legal/immigration status, arranged travel services and/or offered food at no cost, (98, 114, 119) and employed racialized healthcare staff for program delivery (119). The majority of interventions were in publicly funded healthcare settings regardless of country.

### Patient navigation

Eleven studies reported use of navigation to assist underserved women to obtain a mammogram and navigate the health system when an abnormal finding was detected (52, 69, 75, 99), assist women who self-identified as having barriers to care to navigate the health system after receiving an abnormal cervical cancer screening result (88), assist patients to obtain colorectal cancer screening (55, 58), and help patients following cancer diagnosis to gain access to care (56, 57, 29). Navigators were either nurses (75), people with high school diploma (57, 69), or patients who had received training through national and local navigation programs. These interventions/programs employed bilingual navigators or translators and were designed to be culturally sensitive, especially when serving specific racialized groups like Latinos/as and Spanish speaking individuals (52, 58, 69, 89) and Vietnamese American women (99). Study findings suggested that patients were generally satisfied with navigators and services provided (52, 56, 75, 89, 99). They reported that navigation increased the rate of mammography and colorectal cancer screening (52, 55, 58), had a positive impact on the communication between patients, navigators and healthcare providers (58, 69, 88, 89, 99) decreased time from diagnosis to treatment (92, 57, 69, 89), and resulted in decreased numbers of missed appointments (88, 99). Notwithstanding, cultural beliefs about breast cancer and difficulties with reaching patients by phone for initial navigation appointments and follow-ups were identified as barriers in the navigation process (57, 58, 88).

### Education and counselling

Education and counselling interventions were reported in 7 studies. They were used to increase awareness and therefore uptake of breast, cervical and oral cancer screening services for Chinese American (72), African American (95), Black women (73), and people experiencing precarious socioeconomic conditions or those who were uninsured (74, 91). Delivery was either by healthcare providers (91, 120) community health educators, faith-based group leaders, or women’s social organizations (73, 74). These interventions/programs were designed to be flexible, supportive, culturally appropriate and interactive (91, 120, 72, 73, 95). These characteristics were reflected in the availability of pre-booking and/or walk-ins options, onsite screening services, onsite childcare services, transportation cost (72, 73, 91), posters, flyers and videos with culturally appropriate philosophy, content, graphics and language (72, 73), and the provision of opportunities for questions, discussions and hands on demonstration of skills (72, 73, 91, 95). In order to enhance participant’s learning, teaching resources were developed at a low reading level, such as grade 5 (120) and teaching was done in the first language of participants (72). The effectiveness of education and counselling was assessed through retrospective program data (72–74, 95, 120) and pre- and post-intervention mammogram completion questionnaires (72, 74, 91, 95). Studies reported: increase in participants’ cancer awareness and cancer screening for various types of cancer (73, 91), increase in mammography screening rates (72, 73, 95) and feasibility, acceptability and satisfaction with educational programs (73, 91).

### Virtual health interventions/programs

Virtual health interventions were reported in 14 studies and generally targeted rural populations to improve access to specialized care. Mobile phone applications were used to assist patients to self-order screening tests and receive follow-up messages (53) and to send cancer screening reminders (107), while video conferencing employed interactions for genetic counselling (66, 94), specialized cancer care provision (86, 115, 118), chemotherapy supervision (46, 83, 84), exercise-oncology (67), and palliative care (85). They were used to reach as many as 6–63 remote sites (83, 84, 115), 193–254 km away (46, 118).

Video conferencing strategies involved collaboration and supervision between specialised staff in cancer centres and primary healthcare professionals. For example, there were reports of rural generalist nurses administering chemotherapy and biologic therapy agents under the direct supervision of chemotherapy nurses from larger primary centres, using a telenursing platform (46, 83, 84). The uptake of virtual health tools was enhanced by sending messages to patients to remind them of screening schedules (107), sending prescriptions electronically to remote sites or via mail directly to the patient (83, 84), enhancing competencies of remote site staff (67, 84), and providing outreach clinics for patients to see their oncologist in-person (46, 115).

These interventions were assessed using data from retrospective chart reviews (83, 84, 115), and surveys (50, 53, 66). Key outcomes were increased access to cancer care (86, 115), with patients seen and managed locally, within 24 h of referral (83) and patient satisfaction (50, 66, 85, 94). There were significant improvements in screening uptake and adherence (53, 94) and reduction in travel time and cost of care (66, 67, 86, 115). One study reported that patients saved up to 4 h and 40 min on average, 534 km round trip and about $333,074 from lost wages and mileage reimbursement (118). There were also reports of feelings of independence from patients as they received care in their hometown which reduced their reliance on family, friends and government services for transportation (51).

### Service redesign

Service redesign was reported in 10 studies, and entailed modifying or creating innovative healthcare service delivery models to address workforce and geographical accessibility issues. Redesign served to accommodate the cancer care needs of underserved populations, especially in primary healthcare settings. Supervision, education and government support were vital to service redesign (59, 96). Services were redesigned in three main ways: developing capacity of primary healthcare staff to provide specialized services (59, 101), embedding specialized professional or services into primary healthcare packages (71, 76, 93, 96, 108, 113, 116, 117) and developing intersectoral or interdisciplinary collaborations to facilitate care provision (70). Some examples were a nurse-managed health centre that provided breast health services (101), a long-term palliative care unit designed as a hybrid between a hospice and a nursing home (93), and developing a cross-agency collaboration between public health and corrections to provide annual colorectal cancer screening service in a state prison using FIT (70).

Successful service redesign was dependent on capacity building for primary healthcare providers and provision of financial resources by government (59, 96). These services were mainly evaluated via retrospective analyses of routinely gathered data (93, 96, 101, 116, 117). Some reported outcomes were: significant improvement in overall survival and access to care (116, 70, 113, 59, 108), reduction in driving time to medical oncology care, from 51.6 to 19.2 min, with the use of visiting consultant clinics (117), and reduction in treatment delays and increased adherence to treatment and toxicity management (71).

### Financial support

This strategy involved no-cost access to cancer services for people living in poverty or uninsured. They included elimination of copayment, coinsurance, and deductible fees for screening mammography for all women 40 years or older (92, 106), providing free colonoscopy screening to uninsured, asymptomatic patients aged 50–64 years and living below the poverty line (92, 105), and developing a student-run free clinic to provide breast cancer screening opportunities for uninsured patients (104).

These were evaluated using retrospective analyses of service data by either assessing uptake during the intervention period or by comparing trends in screening before and after intervention for periods ranging from 1 to 13 years. Reported outcomes were significant increase in mammography screening rates (106), with 84% of intervention group participants having a mammography post intervention (104), significant increase in colorectal cancer screening, with 79% of participants completing a colonoscopy (105), increased access to timely treatment and improved survival for breast cancer patients (82, 92).

### Reducing geographical barriers

Four studies were specifically aimed at reducing geographical barriers to cancer care in three ways: mobile services, satellite services, and mailouts. Satellite services were designed to provide a similar quality of service as would be found at the main centre (47), while mailouts provided free postal services with paid self-addressed envelopes for return of samples as well as assistance, across the continuum for patients with a positive test (54). Some examples of these interventions were: a mobile, no-cost breast cancer screening program that provided a free screening service to medically underserved women (109); a satellite chemotherapy infusion centre in rural communities where visiting oncology nurse practitioners and/or oncologists from a large urban cancer centre offered chemotherapy services to rural communities (47), and a mailout service where a FIT was mailed to asymptomatic, average-risk veterans overdue for colorectal screening (54). Nurses played a key role in the delivery of the interventions (68, 109). This included performing symptom assessment (68), clinical breast exam (109), and offering chemotherapy services (47).

Intervention evaluation data were collected using questionnaires administered to both patients (54, 68) and healthcare providers (47) or retrospective program data (109). They evaluated participants’ uptake of screening services post intervention (54, 109) and influence of geographical location on screening and screening adherence (68). Studies reported up to 90% screening rates (54) and adherence to screening (68) and participants’ satisfaction with services.

### Multicomponent interventions/programs

Twenty-two studies reported use of a combination of the six categories described above to improve access to cancer care. This ranged from a combination of 2–4 interventions, with education, navigation and financial support being core components of most multicomponent interventions/programs. An example included using a combination of education, financial and geographical accessibility strategies where members of the healthcare team were educated on colorectal cancer screening and ways to improve access, patients had assistance obtaining insurance approvals, and patients who were homebound or unhoused were supplied test kits via a mobile unit (77).

These interventions reported increased access to screening mammography (64, 80, 121), with more than 90% of study participants undertaking screening post intervention (79, 97) and increased access to colorectal cancer screening (60, 61, 65, 77), cervical cancer screening (100, 102) and screening for other types of cancers (103, 114). There were also reports of increased access to psychosocial care, (81) survivorship (98), and patient satisfaction (48).

### Interventions in Canada

Three main interventions were employed by the 6 Canadian studies: virtual health, education and navigation. They were designed specifically for rural populations and Blacks and conducted in Alberta (49, 85), Ontario (73, 119) and British Columbia (115). One study used multiple sites in Alberta, Nova Scotia, and Ontario (67). Virtual health interventions were used to provide palliative care consults (85) and thoracic surgical care (115) to rural and remote patients, and videoconferencing was employed to deliver an exercise oncology program (67). These interventions were found to reduce travel distance and cost and expand access to specialized care for many rural and remote patients as well as improve patient satisfaction. Educational interventions (73), also used in combination with virtual health (119), focused on increasing awareness about breast, cervical and colorectal cancer screening among Blacks and immigrant populations. These studies reported significant increase in cancer screening participation for eligible patients as well as increased awareness of cancer susceptibility and screening guidelines, and improvements in screening self-efficacy. In Alberta, a province-wide standard navigation program was designed for rural Albertans (49). Participants reported a positive impact on their experiences of cancer care, and that accessing a navigator made a difference (49). There was a decrease in visits to the emergency rooms or hospital admissions for cancer-related symptoms, improvements in continuity of care, patient’s ability to access cancer information and meaningful support as well as improved satisfaction with care.

## Discussion

This review explored interventions and programs to improve access to cancer care in underserved populations in high income countries with UHC and the US. Most studies were published between 2018 and 2023 and conducted in the US. Interventions targeted specific access barriers resulting from geographical distance, finances, culture and language, knowledge and health literacy, and health system navigation. Most studies were conducted in community-based settings and recruited participants from faith- and community-based organizations. Participants included underserved women or men, rural populations, and people experiencing low socioeconomic status, incarceration, or multiple social disadvantages. Over two-thirds of interventions and programs focused on access to cancer screening and diagnosis.

Our findings suggest tailored interventions or programs can improve access to cancer care in underserved populations, especially at the screening and diagnosis stage. However, interventions to improve access to cancer treatment, survivorship and palliative/end-of-life care with underserved populations are needed. Most interventions or programs integrated supports to mitigate language, financial, cultural, and geographical barriers. Addressing barriers that prevent underserved populations from timely accessing cancer care is an essential consideration in the delivery of tailored interventions.

Our review findings suggest virtual health interventions and those reducing geographical distance can improve access to screening and diagnosis, oncology treatments (e.g., chemotherapy), supportive care, patient satisfaction as well as reduce travel distance and cost. There were 14 studies that employed virtual health while four focused on reducing geographical distance targeting rural and remote populations and people from low socioeconomic status. Telehealth was the most common intervention (videoconferencing, phone calls) while text or email messages and websites were also utilized. Positive effects of telehealth in cancer care during the COVID-19 pandemic were reported (122). Telehealth may improve access to care for head and neck cancer patients, symptom control and quality of life, and be cost-efficient (123). Findings suggest a need to consider barriers to technology adoption and improve internet access and health literacy (124–126).

Interventions reducing geographical distance included mailing FIT kits, satellite centres, and mobile clinics. A study in Alberta, Canada found that individuals living further from diagnostic facilities had higher odds of no record of colorectal cancer screening (CRC) and people from rural and remote areas had higher odds of being overdue for CRC (127). The study also found that people with higher levels of material deprivation tended to have lower rates of CRC compared to those with lower material deprivation (127). Another Canadian study found that living more than 1-h driving time from a cancer centre was associated with worse overall survival and disease-free survival (128). In geographically large countries such as Canada, interventions aimed at reducing geographical distance and increasing accessibility may contribute to improve cancer outcomes in populations living farther away from cancer centres and those from low income areas.

Interventions that improve affordability of cancer care can have a significant impact on access to cancer screening and treatment. There were five studies that implemented some form of financial support by offering services at no cost, covering the cost of screening, eliminating co-payments, or by expanding Medicaid eligibility. The financial burden of cancer care includes costs related to hospital and physician services, diagnostics, medications, caregiving, employment, travel, and inability to save, among others (129). It affects all dimensions of access to healthcare and will lead underserved populations such as racialized and rural people to delay or decline care (129). In Canada, provincial disparities in public coverage of take-home cancer drugs exist with patient co-payments nearing 20% (130). These out-of-pocket expenses can negatively affect patient’s cancer care decisions and access (130). With over two-thirds of Canadians disclosing financial distress when facing a cancer diagnosis, the need for financial distress mitigation interventions as well as federal and provincial policies is urgent (131).

There were 12 studies that reported patient navigation to improve access to screening and diagnosis, treatment, and patient satisfaction in rural, racialized, uninsured, or low socioeconomic status populations. Navigation interventions in this review incorporated education and counselling, coordination of care, addressing barriers to care, psychosocial care, and financial navigation. The majority of studies showed significant improvements in care outcomes such as access to screening and reduced times from diagnosis to treatment. Patient navigators can play a significant role in the cancer care of patients by improving access to care, patient experience, and care coordination (132). Patient navigators in Canada were described as agents of change who improved patients’ health literacy, built partnerships with agencies to address care inequities, built trust with underserved communities and patient’s trust in the healthcare system (133). The success that patient navigation has shown in improving access to cancer care (36), highlights the key role they can play in improving cancer care outcomes in underserved groups.

Education and service redesign interventions had significant impacts on access to screening, cancer treatment and palliative care, and patient satisfaction in racialized, rural, or people from low socioeconomic status. There were seven studies that reported educational interventions and 10 studies focused on service redesign. Education was provided in the language best understood by participants and used sociocultural adapted resources. Education can be an excellent strategy to increase cancer and cancer service awareness among racialized and immigrant populations (134, 135). Service redesign interventions resulted in cost savings and shorter travel distances. The reorganization of oncology multidisciplinary teams resulted in improved access to and quality of care for lung cancer patients in the United Kingdom (136). A lung cancer service redesign initiative in Australia had impacts on the proportion of new referrals seen within 14 days by specialists and documentation of patients presented at multidisciplinary meetings (137). Studies to evaluate the impact of service redesign on access to cancer care are needed. Considering the rapid growth of immigrant populations in Canada and other countries, promoting access to screening and early cancer diagnoses through education and service redesign may contribute to improve cancer care outcomes.

We identified 22 multi-component interventions with education, navigation, and financial support being the most common core components. The interventions identified in this review showed positive effects on access to cancer screening, psychosocial care, survivorship care, and patient satisfaction. Complex health interventions involve multiple components and are designed to address complex health challenges (138, 139). Our review findings suggest multi-component interventions are suitable to tackle disparities in access to cancer care. Understanding the mechanisms underlying change in complex interventions is important to inform decision makers (139). Further research is needed to understand the interactive effects of intervention components as well as those between the intervention and the context in which it takes place.

The contributions of underserved populations as study participants in included studies reflects their interest in participating in initiatives to improve access to cancer care. Our findings suggest that their successful accrual can be achieved via community organizations, places of worship, and local bulletin boards, social media, flyers and leaflets. Patient navigators and translators, culturally tailored recruitment materials, and covering travel and parking costs can be effective ways of recruiting underserved populations in oncology (140, 141). Most interventions in this review were integrated within community settings. This approach has potential to improve coordination and delivery of cancer prevention, diagnostic, treatment and supportive care services for these populations (142, 143). Establishing partnerships with community members can increase participation and acceptability of interventions for racialized groups (144, 145).

The underserved populations most frequently targeted were those of low socioeconomic status, rural populations, and racialized people. In Canada, populations of low socioeconomic status experience significant cancer disparities (13, 21). In contrast, our review yielded no Canadian studies focused on this population group. This finding may reflect a gap in collecting and reporting study population sociodemographic characteristics. We identified four Canadian studies focusing on rural and remote populations that employed education (73), telehealth (67, 85) or navigation (49) to improve access to prevention and screening services, surgery, rehabilitation, and palliative care. Although evidence is limited, this research can inform the design of services for this population group. Lastly, review findings suggest a need for further research to improve access to cancer care among racialized communities. Although there are calls to improve access to cancer care for underserved populations in Canada (37), review findings suggest minimal Canadian evidence in this area.

Review findings point to the need to increase health equity research in access to cancer care in Canada. The US had the largest number of articles. This likely reflects research funding to address cancer disparities as well as requirements to include underrepresented populations in research. We acknowledge that different healthcare systems might influence the applicability of findings to Canada. This calls for national, provincial, and intersectoral efforts to determine priorities in access to cancer care for underserved populations, advocate before government stakeholders, funding bodies and influence the Canadian research agenda in cancer care.

### Implications

Review findings can inform research, practice and education in the area of access to cancer care for underserved populations in Canada and Alberta. There is a need to accelerate health equity research in cancer care to a) generate evidence of barriers in access to cancer care and determine the magnitude of inequities in cancer care; and b) design, implement and evaluate interventions to improve access to cancer care. There is a need to increase cancer-related health equity research funding to achieve these goals. While this review highlights gaps, research questions to address those gaps need to be informed by affected patients, families, and communities as well as those who provide treatment and care. Engagement of these stakeholders supports integration of research and clinical practice and has potential to accelerate the research to outcome/impact pathway. Involvement of clinical and operational teams in research design and execution is likely to increase uptake of research findings in the cancer care realm. Similarly, research is likely to have greater impact if we engage patients, families, and community members throughout the research cycle.

Clinical practice implications include identifying services and programs currently in place that support underserved populations. Incorporating strategies such as education, service redesign, virtual health, navigation, and the provision of financial, transportation, cultural, and language supports may increase awareness of as well as access to cancer care services. Review findings also show the need to incorporate health equity knowledge in the curricula of health professions, increase both health equity and cultural competency of healthcare professionals, and advance knowledge of educational models to work with underserved populations. The work of critical educator Paulo Freire can inform initiatives aimed at fostering social transformation, empowerment, emancipation, and critical awareness of conditions leading to inequities in access to cancer care (146).

### Limitations

We limited our search to the last 10 years and only included articles published in the English language. Although this period likely reflects time where the majority of studies were published, we may have missed important works published prior to the review period. We also focused on countries with healthcare systems similar to the Canadian universal healthcare system with the exception of the United States. Our exclusion criteria may have resulted in leaving out studies in other countries reporting health equity interventions directed at our populations of interest. Underserved populations comprise a large and diverse group. Our review focused on selected underserved groups. Studies yielding evidence concerning other underserved groups may exist. Estimating the effectiveness of included interventions and their comparative impacts was beyond the scope of this review. We are confident that the breath and recent nature of the studies included provide a current and comprehensive list of interventions tailored to address specific barriers in access to cancer care. This knowledge may inform cancer system stakeholders in the design of programs to support underserved populations facing specific obstacles in access to cancer care such as distance, lack of cancer awareness and health literacy, language and cultural barriers, financial constraints, or health system navigation challenges.

## Conclusion

This systematic review yielded evidence of a wide range of interventions and programs to improve access to cancer care for underserved populations. Utilizing diverse strategies to reach underserved populations and increase intervention uptake is necessary. Review findings suggest these interventions can have a significant impact on patient experiences, satisfaction, and cancer outcomes. Although the majority of interventions were conducted in the US, there is potential to incorporate knowledge from those studies into the Alberta and Canadian cancer care systems. The interventions and programs identified in this review reveal a collective and committed effort to tackle cancer inequities. This is a critical step towards achieving equity in cancer care.

## Data Availability

The original contributions presented in the study are included in the article/Supplementary Material, further inquiries can be directed to the corresponding author.

## References

[1] Vaccarella S Lortet-Tieulent J Saracci R Conway DI Straif K Wild CP . Reducing social inequalities in cancer: evidence and priorities for research. IARC Scientific Publication (2019). No 168. 33443989

[2] Sung H Ferlay J Siegel RL Laversanne M Soerjomataram I Jemal A Global cancer statistics 2020: GLOBOCAN estimates of incidence and mortality worldwide for 36 cancers in 185 countries. CA: A Cancer J Clinicians (2021) 71(3):209–49. 10.3322/caac.21660 33538338

[3] Canadian Cancer Society. Cancer statistics at a glance (2024). Available at: https://cancer.ca/en/research/cancer-statistics/cancer-statistics-at-a-glance#:∼:text=Researchers%20estimated%20that%20there%20would,for%2028.2%25%20of%20all%20deaths (Accessed July 15, 2024).

[4] Canadian Cancer Statistics. Canadian cancer statistics advisory, in collaboration with the Canadian cancer society, statistics Canada, the public health agency of Canada. Toronto, ON: Canadian Cancer Statistics (2023).

[5] Canadian Cancer Statistics. Canadian cancer statistics advisory committee, Canadian cancer society, statistics Canada, public health agency of Canada. Toronto, ON: Canadian cancer statistics (2021).

[6] Canadian Cancer Statistics. Canadian Cancer Statistics: a 2022 special report on cancer prevalence. Health Promot chronic Dis Prev Can (2023) 43(1):49. 10.24095/hpcdp.43.1.05 36651886 PMC9894294

[7] Islami F Guerra CE Minihan A Yabroff KR Fedewa SA Sloan K American Cancer Society's report on the status of cancer disparities in the United States, 2021. CA: A Cancer J Clinicians (2022) 72(2):112–43. 10.3322/caac.21703 34878180

[8] Miller KD Ortiz AP Pinheiro PS Bandi P Minihan A Fuchs HE Cancer statistics for the US Hispanic/Latino population, 2021. CA: A Cancer J Clinicians (2021) 71(6):466–87. 10.3322/caac.21695 34545941

[9] Williams PA Zaidi SK Sengupta R . AACR cancer disparities progress report 2022. Cancer Epidemiol Biomarkers and Prev (2022) 31(7):1249–50. 10.1158/1055-9965.epi-22-0542 35675281

[10] Crowley R Daniel H Cooney TG Engel LS . Envisioning a better U.S. Health care system for all: coverage and cost of care. Ann Intern Med (2020) 172(2 Suppl. l):S7–s32. 10.7326/m19-2415 31958805

[11] Asaria M Ali S Doran T Ferguson B Fleetcroft R Goddard M How a universal health system reduces inequalities: lessons from England. J Epidemiol Community Health (2016) 70(7):637–43. 10.1136/jech-2015-206742 26787198 PMC4941190

[12] Veugelers PJ Yip AM . Socioeconomic disparities in health care use: does universal coverage reduce inequalities in health? J Epidemiol Community Health (2003) 57(6):424–8. 10.1136/jech.57.6.424 12775787 PMC1732477

[13] Canadian Partnership Against Cancer. Examining disparities in cancer control: a system performance special focus report. Toronto, ON: The Canadian Partnership Against Cancer (2014). p. 83.

[14] Canadian Partnership Against Cancer. Cancer system performance: 2017 report. Toronto, ON: The Canadian Partnership Against Cancer (2017).

[15] Dabbikeh A Peng Y Mackillop WJ Booth CM Zhang-Salomons J . Temporal trends in the association between socioeconomic status and cancer survival in Ontario: a population-based retrospective study. CMAJ open (2017) 5(3):E682–E689. 10.9778/cmajo.20170025 PMC5621958 28877916

[16] Truant TLO Lambert LK Thorne S . Barriers to equity in cancer survivorship care: perspectives of cancer survivors and system stakeholders. Glob Qual Nurs Res (2021) 8:233339362110067. 10.1177/23333936211006703 PMC8050754 33912623

[17] Canadian Partnership Against Cancer. The 2018 cancer system performance report. May 6, 2019 (2018). p. 1–63.

[18] Erickson B Biron VL Zhang H Seikaly H Côté DWJ . Survival outcomes of First Nations patients with oral cavity squamous cell carcinoma (Poliquin 2014). Article. J Otolaryngol - Head Neck Surg (2015) 44:2. 10.1186/s40463-015-0056-8 25645260 PMC4323206

[19] Canadian Partnership Against Cancer. The 2016 cancer system performance report. Toronto, ON: Canadian Partnership Against Cancer (2016). p. 128. Available at: http://www.systemperformance.ca/report/2016-cancer-system-performance-report/ (Accessed May 11, 2019).

[20] Public Health Agency of Canada. Key health inequalities in Canada: a national portrait. Ottawa, ON: Pan Canadian Health Inequalities Reporting Initiative (2018).

[21] Canadian Partnership Against Cancer. Lung cancer and equity report (2020). Available at: https://www.partnershipagainstcancer.ca/topics/lung-cancer-equity/ (Accessed July 18, 2022).

[22] Canadian Cancer Statistics Advisory Committee. Canadian Cancer Statistics: a 2020 special report on lung cancer (2020). Available at: https://cancer.ca/canadian-cancer-ctatistics-2020-EN (Accessed July 14, 2021).

[23] Simkin J Woods R Elliott C . Cancer mortality in Yukon 1999-2013: elevated mortality rates and a unique cancer profile. Int J Circumpolar Health (2017) 76(1):1324231. 10.1080/22423982.2017.1324231 28598269 PMC5497549

[24] Iqbal J Ginsburg O Fischer HD Austin PC Creatore MI Narod SA A population-based cross-sectional study comparing breast cancer stage at diagnosis between immigrant and canadian-born women in Ontario. Breast J (2017) 23(5):525–36. 10.1111/tbj.12785 28252245

[25] Lofters AK McBride ML Li D Whitehead M Moineddin R Jiang L Disparities in breast cancer diagnosis for immigrant women in Ontario and BC: results from the CanIMPACT study. journal article. BMC Cancer (2019) 19(1):42. 10.1186/s12885-018-5201-0 30626375 PMC6327524

[26] Vahabi M Lofters A Kumar M Glazier RH . Breast cancer screening disparities among immigrant women by world region of origin: a population-based study in Ontario, Canada. Cancer Med (2016) 5(7):1670–86. 10.1002/cam4.700 27105926 PMC4944895

[27] World Health Organization. Research for universal health coverage (2013). Available at: https://www.afro.who.int/publications/world-health-report-2013-research-universal-health-coverage (Accessed May 29, 2023).

[28] United Nations Department of Economic and Social Affairs Sustainable Development. Goal 3 Ensure healthy lives and promote well-being for all at all ages (2024). Available at: https://sdgs.un.org/goals/goal3 (Accessed May 14, 2024).

[29] Martin D Miller AP Quesnel-Vallée A Caron NR Vissandjée B Marchildon GP . Canada's universal health-care system: achieving its potential. The Lancet (2018) 391(10131):1718–35. 10.1016/s0140-6736(18)30181-8 PMC7138369 29483027

[30] Canadian Cancer Society. Advancing health equity through cancer information and support services (2023). Report on communities that are underserved.

[31] Alberta Cancer Strategic Clinical Network. Future of cancer impact in Alberta. Alberta, Canada (2023).

[32] Patel M Andrea N Jay B Coker TR . A community-partnered, evidence-based approach to improving cancer care delivery for low-income and minority patients with cancer. J Community Health (2019) 44(5):912–20. 10.1007/s10900-019-00632-x 30825097 PMC7046315

[33] Maliski SL Clerkin B Litwin MS . Describing a nurse case manager intervention to empower low-income men with prostate cancer. Oncol Nurs Forum (2004) 31(1):57–64. 10.1188/04.onf.57-64 14722588

[34] Bergman J Chi AC Litwin MS . Quality of end-of-life care in low-income, uninsured men dying of prostate cancer. Cancer (2010) 116(9):2126–31. 10.1002/cncr.25039 20198706

[35] Simon MA Nonzee NJ McKoy JM Liu D Luu TH Byer P Navigating veterans with an abnormal prostate cancer screening test: a quasi-experimental study. BMC Health Serv Res (2013) 13:314. 10.1186/1472-6963-13-314 23947435 PMC3844412

[36] Chan RJ Milch VE Crawford-Williams F Agbejule OA Joseph R Johal J Patient navigation across the cancer care continuum: an overview of systematic reviews and emerging literature. CA: A Cancer J Clinicians (2023) 73(6):565–89. 10.3322/caac.21788 37358040

[37] Canadian Partnership Against Cancer. 2019-2029 Canadian strategy for cancer control (2019).

[38] Rajaguru V Jang J Kwon JA Kim JH Shin J Chun M . A scoping review on population-centered indicators for cancer care continuum. Front Public Health (2022) 10:912946. 10.3389/fpubh.2022.912946 36311597 PMC9614426

[39] Scanlon B Brough M Wyld D Durham J . Equity across the cancer care continuum for culturally and linguistically diverse migrants living in Australia: a scoping review. Glob Health (2021) 17(1):87. 10.1186/s12992-021-00737-w PMC8318324 34321015

[40] Levesque JF Harris MF Russell G . Patient-centred access to health care: conceptualising access at the interface of health systems and populations. Int J Equity Health (2013) 12:18. 10.1186/1475-9276-12-18 23496984 PMC3610159

[41] Page MJ Moher D Bossuyt PM Boutron I Hoffmann TC Mulrow CD PRISMA 2020 explanation and elaboration: updated guidance and exemplars for reporting systematic reviews. BMJ (2021) 372:n160. 10.1136/bmj.n160 33781993 PMC8005925

[42] Cochrane Handbook. Cochrane Handbook (2024). Available at: https://training.cochrane.org/handbook/current (Accessed February 12, 2024).

[43] Effective Public Healthcare Panacea Project. Quality assessment tool for quantitative studies (2023). Available at: https://www.ephpp.ca/quality-assessment-tool-for-quantitative-studies/(Accessed May 4, 2023).

[44] Critical Appraisal Skills Programme. CASP qualitative studies checklist (2023). Available at: https://casp-uk.net/images/checklist/documents/CASP-Qualitative-Studies-Checklist/CASP-Qualitative-Checklist-2018_fillable_form.pdf (Accessed May 4, 2023).

[45] Kiger ME Varpio L . Thematic analysis of qualitative data: AMEE Guide No. 131. Med Teach (2020) 42(8):846–54. 10.1080/0142159x.2020.1755030 32356468

[46] Pye S Webster E Zielinski R Honeyball F . The best thing since sliced bread': patient experiences of teleoncology in western NSW. Aust J Rural Health (2023) 31(1):90–7. 10.1111/ajr.12921 36053275

[47] Curtis ML Eschiti VS . Geographic health disparities: satellite clinics for cancer care in rural communities. Clin J Oncol Nurs (2018) 22(5):500–6. 10.1188/18.Cjon.500-506 30239508

[48] Foley J Ward EC Burns CL Nund RL Wishart LR Graham N Enhancing speech-language pathology head and neck cancer service provision in rural Australia: using a plan, do, study, act approach. Int J Speech-Language Pathol (2023) 25(2):292–305. 10.1080/17549507.2022.2050300 35532005

[49] Watson LC Vimy K Anderson J Champ S DeIure A . Developing a provincial cancer patient navigation program utilizing a quality improvement approach. Part three: evaluation and outcomes. Can Oncol Nurs J (2016) 26(4):276–85. 10.5737/23688076264276285 31148676 PMC6516265

[50] Arnold JL Anderson E Roeder L . Rural patients highly satisfied with gynaecological oncology care via telehealth. Aust New Zealand J Obstet Gynaecol (2022) 62(2):280–5. 10.1111/ajo.13452 34713445

[51] Fennell KM Turnbull DA Bidargaddi N McWha JL Davies M Olver I . The consumer-driven development and acceptability testing of a website designed to connect rural cancer patients and their families, carers and health professionals with appropriate information and psychosocial support. Eur J Cancer Care (Engl) (2017) 26(5):e12533. 10.1111/ecc.12533 27405399

[52] Li Y Carlson E Hernández DA Green B Calle T Kumaresan T Patient perception and cost-effectiveness of a patient navigation program to improve breast cancer screening for hispanic women. Health Equity (2019) 3(1):280–6. 10.1089/heq.2018.0089 31236527 PMC6588102

[53] Denizard-Thompson NM Miller DP Snavely AC Spangler JG Case LD Weaver KE . Effect of a digital health intervention on decreasing barriers and increasing facilitators for colorectal cancer screening in vulnerable patients. Cancer Epidemiol Biomarkers and Prev (2020) 29(8):1564–9. 10.1158/1055-9965.Epi-19-1199 PMC7416430 32381556

[54] Charlton ME Mengeling MA Halfdanarson TR Makki NM Malhotra A Klutts JS Evaluation of a home-based colorectal cancer screening intervention in a rural state. The J Rural Health (2014) 30(3):322–32. 10.1111/jrh.12052 24164375 PMC4266988

[55] Menon U Szalacha LA Kue J Herman PM Bucho-Gonzalez J Lance P Effects of a community-to-clinic navigation intervention on colorectal cancer screening among underserved people. Ann Behav Med (2020) 54(5):308–19. 10.1093/abm/kaz049 31676898 PMC7168576

[56] Battaglia TA Gunn CM Bak SM Flacks J Nelson KP Wang N Patient navigation to address sociolegal barriers for patients with cancer: a comparative-effectiveness study. Cancer (2022) 128(Suppl. 13):2623–35. 10.1002/cncr.33965 35699610 PMC10152516

[57] Freund KM Battaglia TA Calhoun E Darnell JS Dudley DJ Fiscella K Impact of patient navigation on timely cancer care: the Patient Navigation Research Program. J Natl Cancer Inst (2014) 106(6):dju115. 10.1093/jnci/dju115 24938303 PMC4072900

[58] Percac-Lima S López L Ashburner JM Green AR Atlas SJ . The longitudinal impact of patient navigation on equity in colorectal cancer screening in a large primary care network. Cancer (2014) 120(13):2025–31. 10.1002/cncr.28682 24691564

[59] Hall F Gordon S Hulcombe J Stephens C . Compression garment service model: facilitating access to compression garments through workforce and service redesign. Aust J Rural Health (2019) 27(3):257–61. 10.1111/ajr.12509 31070819

[60] Baker DW Brown T Buchanan DR Weil J Balsley K Ranalli L Comparative effectiveness of a multifaceted intervention to improve adherence to annual colorectal cancer screening in community health centers: a randomized clinical trial. JAMA Intern Med (2014) 174(8):1235–41. 10.1001/jamainternmed.2014.2352 24934845

[61] Davis TC Rademaker A Morris J Ferguson LA Wiltz G Arnold CL . Repeat annual colorectal cancer screening in rural community clinics: a randomized clinical trial to evaluate outreach strategies to sustain screening. The J Rural Health (2020) 36(3):307–15. 10.1111/jrh.12399 31523848 PMC9396927

[62] Champion VL Paskett ED Stump TE Biederman EB Vachon E Katz ML Comparative effectiveness of 2 interventions to increase breast, cervical, and colorectal cancer screening among women in the rural US: a randomized clinical trial. JAMA Netw Open (2023) 6(4):e2311004. 10.1001/jamanetworkopen.2023.11004 37115541 PMC10148202

[63] Fang ML Sixsmith J Sinclair S Horst G . A knowledge synthesis of culturally- and spiritually-sensitive end-of-life care: findings from a scoping review. BMC Geriatr (2016) 16:107. 10.1186/s12877-016-0282-6 27193395 PMC4872365

[64] Hendren S Winters P Humiston S Idris A Li SXL Ford P Randomized, controlled trial of a multimodal intervention to improve cancer screening rates in a safety-net primary care practice. J Gen Intern Med (2014) 29(1):41–9. 10.1007/s11606-013-2506-1 23818159 PMC3889982

[65] Manning M Lucas T Thompson H Penner L . Results of an African American-targeted norm-based colorectal cancer screening intervention: a pilot study. J Behav Med (2023) 46(3):391–404. 10.1007/s10865-022-00367-6 36205850

[66] Solomons NM Lamb AE Lucas FL McDonald EF Miesfeldt S . Examination of the patient-focused impact of cancer telegenetics among a rural population: comparison with traditional in-person services. Telemed e-Health (2018) 24(2):130–8. 10.1089/tmj.2017.0073 PMC8259038 28737998

[67] Wagoner CW Dreger J Keats MR Santa Mina D McNeely ML Cuthbert C First-year implementation of the EXercise for cancer to enhance living well (EXCEL) study: building networks to support rural and remote community access to exercise oncology resources. Int J Environ Res Public Health (2023) 20(3):1930. 10.3390/ijerph20031930 36767296 PMC9915392

[68] Balata H Tonge J Barber PV Colligan D Elton P Evison M Attendees of Manchester's Lung Health Check pilot express a preference for community-based lung cancer screening. Thorax (2019) 74(12):1176–8. 10.1136/thoraxjnl-2018-212601 31481631

[69] Ramirez A Perez-Stable E Penedo F Talavera G Carrillo JE Fernández M Reducing time-to-treatment in underserved latinas with breast cancer: the six cities study. Cancer (2014) 120(5):752–60. 10.1002/cncr.28450 24222098 PMC3949173

[70] Dumont DM Davis D Sadacharan R Lamy E Clarke JG . A correctional-public health collaboration for colorectal cancer screening in a state prison system. Public Health Rep (2021) 136(5):548–53. 10.1177/0033354920974668 33563069 PMC8361559

[71] Patel JV Hughes DM Ko NY . OPTIMAL breast cancer care: effect of an outpatient pharmacy team to improve management and adherence to oral cancer treatment. JCO Oncol Pract (2023) 19(3):e306–e314. 10.1200/op.22.00135 36480784

[72] Lee-Lin F Menon U Leo MC Pedhiwala N . Feasibility of a targeted breast health education intervention for Chinese American immigrant women. Oncol Nurs Forum (2013) 40(4):361–72. 10.1188/13.Onf.361-372 23803269

[73] Lofters A Jain A Siu W Kyte M Lee-Foon N Scott F Ko-Pamoja: the feasibility of a lay health educator-led breast and cervical screening program for Black women in Ontario, Canada (short report). Cancer Causes Control (2017) 28(11):1207–18. 10.1007/s10552-017-0920-0 28685277

[74] Niranjan SJ Opoku-Agyeman W Hardy CM Bowman T Vedre-Kyanam A Hearld KR Using community health advisors to increase lung cancer screening awareness in the Black belt: a pilot study. J Cancer Education (2023) 38(4):1286–95. 10.1007/s13187-022-02261-w PMC11288175 36650394

[75] Williams MA Nielsen DR Dayao Z Brown-Glaberman U Tawfik B . Patient-reported measures of a breast cancer nurse navigator program in an underserved, rural, and economically disadvantaged patient population. Oncol Nurs Forum (2022) 49(6):532–9. 10.1188/22.Onf.532-539 36413732

[76] Holle LM Levine J Buckley T White CM White C Hadfield MJ . Pharmacist intervention in colorectal cancer screening initiative. J Am Pharm Assoc (2003) (2020) 60(4):e109–e116. 10.1016/j.japh.2020.02.014 32197754

[77] Centra T Fogg C . Addressing barriers to colorectal cancer screening in a federally qualified health center. J Am Assoc Nurse Pract (2023) 35(7):415–24. 10.1097/jxx.0000000000000828 36745041

[78] Kim KE Tangka FKL Jayaprakash M Randal FT Lam H Freedman D Effectiveness and cost of implementing evidence-based interventions to increase colorectal cancer screening among an underserved population in chicago. Health Promotion Pract (2020) 21(6):884–90. 10.1177/1524839920954162 PMC7894065 32990041

[79] Molokwu JC Dwivedi A Alomari A Shokar N . Effectiveness of a breast cancer education screening and NavigaTion (BEST) intervention among hispanic women. Health Promotion Pract (2023):15248399221135762. 10.1177/15248399221135762 36635866

[80] Richman AR Torres E Wu Q Kampschroeder AP . Evaluating a community-based breast cancer prevention program for rural underserved latina and Black women. J Community Health (2020) 45(6):1205–10. 10.1007/s10900-020-00856-2 32529466

[81] Azizoddin DR Lakin JR Hauser J Rynar LZ Weldon C Molokie R Meeting the guidelines: implementing a distress screening intervention for veterans with cancer. Psychooncology (2020) 29(12):2067–74. 10.1002/pon.5565 33009712

[82] Rajan SS Begley CE Highfield LD Kim B . Survival benefits of treatment access among underserved breast cancer patients diagnosed through the Texas breast and cervical cancer services program. J Public Health Management Pract (2015) 21(5):477–86. 10.1097/phh.0000000000000255 25794245

[83] Sabesan S Roberts LJ Aiken P Joshi A Larkins S . Timely access to specialist medical oncology services closer to home for rural patients: experience from the Townsville Teleoncology Model. Aust J Rural Health (2014) 22(4):156–9. 10.1111/ajr.12101 25123618

[84] Sabesan S Senko C Schmidt A Joshi A Pandey R Ryan CA Enhancing chemotherapy capabilities in rural hospitals: implementation of a telechemotherapy model (QReCS) in north queensland, Australia. J Oncol Pract (2018) 14(7):e429–e437. 10.1200/jop.18.00110 29996068

[85] Watanabe SM Fairchild A Pituskin E Borgersen P Hanson J Fassbender K . Improving access to specialist multidisciplinary palliative care consultation for rural cancer patients by videoconferencing: report of a pilot project. Support Care Cancer (2013) 21(4):1201–7. 10.1007/s00520-012-1649-7 23161339

[86] Hitt WC Low GM Lynch CE Gauss CH Magann EF Lowery CL Application of a telecolposcopy program in rural settings. Telemed e-Health (2016) 22(10):816–20. 10.1089/tmj.2015.0260 27128600

[87] Drake B James A Miller H Anandarajah A Davis KL Jackson S Strategies to achieve breast health equity in the St. Louis region and beyond over 15+ years. Cancers (Basel) (2022) 14(10):2550. 10.3390/cancers14102550 35626157 PMC9140077

[88] Luckett R Pena N Vitonis A Bernstein MR Feldman S . Effect of patient navigator program on no-show rates at an academic referral colposcopy clinic. J Women's Health (2015) 24(7):608–15. 10.1089/jwh.2014.5111 26173000

[89] Vilchis H Onstad LE Benavidez R Castillo R Bush N Sanchez J Una mano amiga: pilot test of a patient navigator program for southwest New Mexico. J Cancer Education (2019) 34(1):173–9. 10.1007/s13187-017-1283-7 PMC5871533 28956318

[90] Lara CL Means KL Morwood KD Lighthall WR Hoover S Tangka FK Colorectal cancer screening interventions in 2 health care systems serving disadvantaged populations: screening uptake and cost-effectiveness. Cancer (2018) 124(21):4130–6. 10.1002/cncr.31691 30359479 PMC6263828

[91] Williams MS Wells J Duhé RJ Shirley T Lampkin A Murphy M The college of American pathologists foundation’s see, test and treat Program®: an evaluation of a one-day cancer screening program implemented in Mississippi. J Cancer Education (2022) 37(6):1912–7. 10.1007/s13187-021-02060-9 34164764

[92] Johnson CJ Morawski BM Hobbs L Lewis D Cariou C Rycroft RK . Time from breast cancer diagnosis to treatment among Idaho's national breast and cervical cancer early detection program population, 2011-2017. Cancer Causes Control (2021) 32(6):667–73. 10.1007/s10552-021-01407-3 33665701

[93] Wakefield D If not home, where? Implementing an innovative model of care as an alternative place of care and death for patients living in an area of high socio-economic deprivation. Short-report on opening a long-term palliative care unit. Palliat Med (2023) 37(4):652–6. 10.1177/02692163221133984 36337044

[94] Mette LA Saldívar AM Poullard NE Torres I Seth S Pollock B Reaching high-risk underserved individuals for cancer genetic counseling by video-teleconferencing. J Community Support Oncol (2016) 14(4):162–8. 10.12788/jcso.0247 27152515

[95] Wilson-Anderson K Williams PR Beacham T McDonald N . Breast health teaching in predominantly African American rural Mississippi Delta. The ABNF J (2013) 24(1):28–33. 23589970

[96] Hoskins KF Tejeda S Vijayasiri G Chukwudozie IB Remo MH Shah HA A feasibility study of breast cancer genetic risk assessment in a federally qualified health center. Cancer (2018) 124(18):3733–41. 10.1002/cncr.31635 30320429 PMC6214782

[97] Mayfield-Johnson S Fastring D Fortune M White-Johnson F . Addressing breast cancer health disparities in the Mississippi delta through an innovative partnership for education, detection, and screening. J Community Health (2016) 41(3):494–501. 10.1007/s10900-015-0121-2 26578349

[98] Sanchez-Birkhead AC Carbajal-Salisbury S Arce Larreta J Hendricks H Beck SL . Addressing disparities: the alliance breast cancer community-based program for hispanic women. Clin J Oncol Nurs (2016) 20(5):481–6. 10.1188/16.Cjon.20-05ap 27668368

[99] Thai CL Ong G Tran T Le Y . Assessing the impact of a patient navigator intervention program for Vietnamese-American women with abnormal mammograms. J Cancer Education (2022) 37(3):621–30. 10.1007/s13187-020-01856-5 32880868

[100] Fang CY Ma GX Handorf EA Feng Z Tan Y Rhee J Addressing multilevel barriers to cervical cancer screening in Korean American women: a randomized trial of a community-based intervention. Cancer (2017) 123(6):1018–26. 10.1002/cncr.30391 27869293 PMC5339039

[101] Tsai PY Peterman B Baisch MJ Ji ES Zwiers K . Providing and funding breast health services in urban nurse-managed health centers. Nurs Outlook (2014) 62(3):204–11. 10.1016/j.outlook.2014.01.001 24739700

[102] Kiser LH Butler J . Improving equitable access to cervical cancer screening and management. AJN, Am J Nurs (2020) 120(11):58–67. 10.1097/01.Naj.0000721944.67166.17 33105224

[103] Limaye N Zorzato D Nadarajasundaram A Ong SBY . Increasing the uptake in a general practice prostate cancer screening programme. BMJ Open Qual (2022) 11(1):e001701. 10.1136/bmjoq-2021-001701 PMC8915375 35272998

[104] Khalil S Hatch L Price CR Palakurty SH Simoneit E Radisic A Addressing breast cancer screening disparities among uninsured and insured patients: a student-run free clinic initiative. J Community Health (2020) 45(3):501–5. 10.1007/s10900-019-00767-x 31667647

[105] Eberth JM Thibault A Caldwell R Josey MJ Qiang B Peña E A statewide program providing colorectal cancer screening to the uninsured of South Carolina. Cancer (2018) 124(9):1912–20. 10.1002/cncr.31250 29415338 PMC5910186

[106] Peppercorn J Horick N Houck K Rabin J Villagra V Lyman GH Impact of the elimination of cost sharing for mammographic breast cancer screening among rural US women: a natural experiment. Cancer (2017) 123(13):2506–15. 10.1002/cncr.30629 28195644

[107] Offman J Myles J Ariyanayagam S Colorado Z Sharp M Cruice M A telephone reminder intervention to improve breast screening information and access. Public Health (2014) 128(11):1017–22. 10.1016/j.puhe.2014.09.007 25443131

[108] Gunness P Hamilton S Capstick R Masters J Toma R . The development of a rural breast reconstruction service: patient reported outcomes and benefits. ANZ J Surg (2023) 93(7-8):1935–7. 10.1111/ans.18389 36944602

[109] van den Bruele AB Sevilimedu V Jochelson M Formenti S Norton L Sacchini V . Mobile mammography in New York City: analysis of 32,350 women utilizing a screening mammogram program. NPJ Breast Cancer (2022) 8(1):14. 10.1038/s41523-022-00381-6 35064104 PMC8782895

[110] Gabitova G Burke NJ . Improving healthcare empowerment through breast cancer patient navigation: a mixed methods evaluation in a safety-net setting. BMC Health Serv Res (2014) 14:407. 10.1186/1472-6963-14-407 25234963 PMC4177686

[111] Li J Cornacchi SD Farrokhyar F Johnston N Forbes S Reid S Relation between socioeconomic variables and surgical, systemic and radiation treatment in a cohort of patients with breast cancer in an urban Canadian centre. Can J Surg (2019) 62(2):83–92. 10.1503/cjs.009217 30697993 PMC6440893

[112] Mema SC Yang H Elnitsky S Jiang Z Vaska M Xu L . Enhancing access to cervical and colorectal cancer screening for women in rural and remote northern Alberta: a pilot study. CMAJ Open (2017) 5(4):E740–e745. 10.9778/cmajo.20170055 PMC5741430 28974533

[113] Galiatsatos P Schreiber R Green K Shah R Lee H Feller-Kopman D Improving lung cancer screening: an equitable strategy through a tobacco treatment clinic. Prev Med Rep (2021) 24:101558. 10.1016/j.pmedr.2021.101558 34976626 PMC8683889

[114] Le T Miller S Berry E Zamarripa S Rodriguez A Barkley B Implementation and uptake of rural lung cancer screening. J Am Coll Radiol (2022) 19(3):480–7. 10.1016/j.jacr.2021.12.003 35143786 PMC8923939

[115] Humer MF Campling BG . The role of telemedicine in providing thoracic oncology care to remote areas of British Columbia. Curr Oncol Rep (2017) 19(8):52. 10.1007/s11912-017-0612-7 28664469

[116] Swayze EJ Strzyzewski L Avula P Zebolsky AL Hoekstra AV . The impact of expanding gynecologic oncology care to ovarian cancer patients in small cities and rural communities. Gynecol Oncol (2021) 161(3):852–7. 10.1016/j.ygyno.2021.04.021 33888339

[117] Tracy R Nam I Gruca TS . The influence of visiting consultant clinics on measures of access to cancer care: evidence from the state of Iowa. Health Serv Res (2013) 48(5):1719–29. 10.1111/1475-6773.12050 23480819 PMC3796110

[118] Thota R Gill DM Brant JL Yeatman TJ Haslem DS . Telehealth is a sustainable population health strategy to lower costs and increase quality of health care in rural Utah. JCO Oncol Pract (2020) 16(7):e557–e562. 10.1200/jop.19.00764 32463765

[119] Nnorom O Sappong-Kumankumah A Olaiya OR Burnett M Akor N Shi N Afrocentric screening program for breast, colorectal, and cervical cancer among immigrant patients in Ontario. Can Fam Physician (2021) 67(11):843–9. 10.46747/cfp.6711843 34772714 PMC8589122

[120] Schroeder K Panny A Shimpi N . Community awareness and oral cancer screening in rural Wisconsin. J Dent Hyg (2021) 95(4):51–8. 34376544

[121] Lane AJ Martin MT . Ten year profile of a best practice program aimed at rural women. Online J Rural Nurs Health Care (2015) 15(2). 10.14574/ojrnhc.v15i2.363

[122] Mostafaei A Sadeghi-Ghyassi F Kabiri N Hajebrahimi S . Experiences of patients and providers while using telemedicine in cancer care during COVID-19 pandemic: a systematic review and meta-synthesis of qualitative literature. Support Care Cancer (2022) 30(12):10483–94. 10.1007/s00520-022-07415-6 36322247 PMC9628519

[123] Caputo MP Rodriguez CS Padhya TA Mifsud MJ . Telehealth interventions in head and neck cancer patients: a systematic review. Cancer Nurs (2023) 46(5):E320–e327. 10.1097/ncc.0000000000001130 37607382

[124] Chesser A Burke A Reyes J Rohrberg T . Navigating the digital divide: a systematic review of eHealth literacy in underserved populations in the United States. Inform Health Social Care (2016) 41(1):1–19. 10.3109/17538157.2014.948171 25710808

[125] Lee EW McCloud RF Viswanath K . Designing effective eHealth interventions for underserved groups: five lessons from a decade of eHealth intervention design and deployment. J Med Internet Res (2022) 24(1):e25419. 10.2196/25419 34994700 PMC8783288

[126] Nagler RH Ramanadhan S Minsky S Viswanath K . Recruitment and retention for community-based eHealth interventions with populations of low socioeconomic position: strategies and challenges. J Commun (2013) 63(1):201–20. 10.1111/jcom.12008 23439871 PMC3579669

[127] Jessiman-Perreault G Law J Adhikari K Machado AA Moysey B Xu L Geospatial analysis and participant characteristics associated with colorectal cancer screening participation in Alberta, Canada: a population-based cross-sectional study. BMC Health Serv Res (2023) 23(1):1454. 10.1186/s12913-023-10486-8 38129826 PMC10740253

[128] Gotfrit J Thangarasa T Dudani S Goodwin R Tang PA Monzon J The impact of driving time, distance, and socioeconomic factors on outcomes of patients with locally advanced rectal cancer. Public Health Pract (Oxford, England) (2020) 1:100012. 10.1016/j.puhip.2020.100012 PMC9461354 36101686

[129] Abrams HR Durbin S Huang CX Johnson SF Nayak RK Zahner GJ Financial toxicity in cancer care: origins, impact, and solutions. Translational Behav Med (2021) 11(11):2043–54. 10.1093/tbm/ibab091 34850932

[130] MacPhail C Snow S . Not all Canadian cancer patients are equal-disparities in public cancer drug funding across Canada. Curr Oncol (2022) 29(3):2064–72. 10.3390/curroncol29030166 35323366 PMC8947051

[131] Wood TF Murphy RA . Tackling financial toxicity related to cancer care in Canada. CMAJ (2024) 196(9):E297–e298. 10.1503/cmaj.230677 38467415 PMC10927292

[132] Canadian Association of Nurses in Oncology. Patient Navigator in cancer care-A specialized oncology nurse role that contributes to high-quality, person-centred care experiences and clinical efficiencies. Can Oncol Nurs J Summer (2020) 30(3):227–8. PMC7583568 33118967

[133] Katerenchuk J Santos Salas A . An integrative review on the oncology nurse navigator role in the Canadian context. Can Oncol Nurs J (2023) 33(4):385–99. 10.5737/23688076334385 38919590 PMC11195828

[134] Ghahari S Burnett S Alexander L . Development and pilot testing of a health education program to improve immigrants' access to Canadian health services. BMC Health Serv Res (2020) 20(1):321. 10.1186/s12913-020-05180-y 32303224 PMC7164356

[135] Dunn SF Lofters AK Ginsburg OM Meaney CA Ahmad F Moravac MC Cervical and breast cancer screening after cares: a community program for immigrant and marginalized women. Am J Prev Med (2017) 52(5):589–97. 10.1016/j.amepre.2016.11.023 28094134

[136] Salem A Bayman N . Multidisciplinary team service redesign: a step to improved quality of care for lung cancer patients. Clin Oncol (2016) 28(12):800–1. 10.1016/j.clon.2016.06.012 27378316

[137] Largey G Briggs P Davies H Underhill C Ross C Harvey K Victorian Lung Cancer Service Redesign Project: impacts of a quality improvement collaborative on timeliness and management in lung cancer. Intern Med J (2021) 51(12):2061–8. 10.1111/imj.15043 32896957

[138] O'Cathain A Croot L Duncan E Rousseau N Sworn K Turner KM Guidance on how to develop complex interventions to improve health and healthcare. BMJ open (2019) 9(8):e029954. 10.1136/bmjopen-2019-029954 PMC6701588 31420394

[139] Skivington K Matthews L Simpson SA Craig P Baird J Blazeby JM A new framework for developing and evaluating complex interventions: update of Medical Research Council guidance. BMJ (2021) 374:n2061. 10.1136/bmj.n2061 34593508 PMC8482308

[140] Cook ED Yeager KA Cecchini RS Boparai J Brown CL Duncan M Recruitment practices for U.S. minority and underserved populations in NRG oncology: results of an online survey. Contemp Clin trials Commun (2018) 10:100–4. 10.1016/j.conctc.2018.03.003 30023443 PMC6046466

[141] Rogers CR Matthews P Brooks E Le Duc N Washington C McKoy A Barriers to and facilitators of recruitment of adult african American men for colorectal cancer research: an instrumental exploratory case study. JCO Oncol Pract (2021) 17(5):e686–e694. 10.1200/op.21.00008 33974818 PMC8258132

[142] Stockman LS Gundersen DA Gikandi A Akindele RN Svoboda L Pohl S The colocation model in community cancer care: a description of patient clinical and demographic attributes and referral pathways. JCO Oncol Pract (2023) 19(6):e916–e926. 10.1200/op.22.00487 36940391 PMC10332843

[143] Winkfield KM Regnante JM Miller-Sonet E González ET Freund KM Doykos PM . Development of an actionable framework to address cancer care disparities in medically underserved populations in the United States: expert roundtable recommendations. JCO Oncol Pract (2021) 17(3):e278–e293. 10.1200/op.20.00630 33464925 PMC8202060

[144] Matsuda Y Brooks JL Beeber LS . Guidelines for research recruitment of underserved populations (EERC). Appl Nurs Res (2016) 32:164–70. 10.1016/j.apnr.2016.07.009 27969022 PMC5215593

[145] Huang B De Vore D Chirinos C Wolf J Low D Willard-Grace R Strategies for recruitment and retention of underrepresented populations with chronic obstructive pulmonary disease for a clinical trial. BMC Med Res Methodol (2019) 19(1):39. 10.1186/s12874-019-0679-y 30791871 PMC6385381

[146] Freire P . Conscientização: teoria e prática da libertação: uma introdução ao pensamento de Paulo Freire [Conscientization: Theory and practice of liberation: an introduction to Paulo Freire's thought]. Cintra Kdmesrtdbel Cortez and Moraes (1987).

