# Supplementary material for: Interventions to improve access to cancer care in underserved populations in high income countries: a systematic review
Source: Oncol Rev. 2024 Nov 5;18:1427441. doi: 10.3389/or.2024.1427441 (PMC11573526; doi:10.3389/or.2024.1427441)
Supplement: Supplementary file 1 [file Table1.pdf]

Interventions to improve access to cancer care in underserved populations in high income countries: A systematic review

Supplementary Table 1: Overview of interventions and outcomes by study

Author and Date	Underserved Group	Key Intervention Components	Study Design	Outcome	Outcome Category
**Virtual health**					
Arnold et al 2022	Rural and remote women	Fortnightly telehealth consultations for gynecological malignancies in a rural clinic by specialist staff from a gynaecologic oncology center.	Mixed methods retrospective survey	Mean satisfaction was 90.5%. Feelings of nervousness or fear were alleviated after consultations.	Patient satisfaction<br><br>Access to cancer treatment
Denizard-Thompson et al 2020	Low SES: rural, African American, low income, low literacy.	A validated brief decision aid about CRC screening, patient self-ordering of tests, and follow-up text or email messages to promote screening test completion.	Randomized controlled trial	Intervention group participants were twice as likely to have CRC screening compared with Control group (30% vs.15%)	Access to screening
Fennell et al 2017	Rural	A website that provides rural-specific information on psychosocial care for cancer patients	Mixed methods participatory action research	Increased patients' motivation and confidence in accessing psychosocial support services (66.67%), less feeling of isolation (73.33%) and/or distressed (53.57%).	Access to psychosocial and supportive care
Hitt et al 2016	Rural women	Tele-colposcopy for women with an abnormal Pap test or abnormal visual examination and who required further evaluation by colposcopy.	Retrospective database analysis	Over a 15-month time period, 940 unique patients were seen using tele-colposcopy.	Access to screening

Humer et al 2017	Rural and remote	Use of telemedicine to provide thoracic surgical care.	Retrospective database analysis	Telemedicine saved a total travel distance of 11.5 million km—an average of 766 km per patient.	Access to cancer treatment
Mette et al 2016	Low SES: rural, uninsured or underinsured	Cancer genetic risk assessment (CGRA) and counseling through telemedicine	Retrospective analysis	Overall high level of satisfaction with the program	Access to genetic counselling (GC)<br><br>Patient satisfaction
Offman et al 2014	High deprivation and ethnic diverse women	Calling women invited for breast screening to confirm receipt of invitation, remind them of upcoming visit, and educate them about benefits and risks.	Retrospective database analysis	A 10% increase in women reached resulted in an 8% increase in the odds of women attending breast cancer screening appointments (95% CI: 5%e11%)	Access to screening<br><br>Patient satisfaction
Pye et al 2023	Rural	Remote video-assisted chemotherapy program providing low- and medium-risk cancer therapies to outpatients. Provided by a ward-based trained chemotherapy nurse, supervised by a specialist chemotherapy nurse.	Qualitative	Patients appreciated the reduced physical and emotional travel burden in addition to reduction in travel time and cost.	Access to cancer treatment<br><br>Patient satisfaction
Sabesan et al 2014	Rural	Oncologists services provided through videoconferencing to rural sites. Medical oncologists write eligible patients' chemotherapy care plan, and send prescriptions electronically to rural sites where it is	Retrospective database analysis	Between 2009 and 2011, cancer care was provided to 70 new patients. Of these new patients, 93% (65/70) were seen within one week of referral. All 17 patients requiring urgent reviews were seen within 24 hours of referral and managed locally.	Access to cancer treatment

		administered by chemotherapy-competent nurses. Oral chemotherapy, prescriptions are sent via post to patients or the pharmacy after appropriate education.			
Sabesan et al 2018	Rural	Rural generalist nurses administer chemotherapy and biologic therapy agents under the direct supervision of chemotherapy-proficient nurses from larger primary centres, using a telenursing platform. Rural generalist medical officers and pharmacists provide local support, and medical oncologists and oncology pharmacists from larger primary centers provide guidance via telehealth	Retrospective database analysis	Sixty-two patients received 327 cycles of low- to medium-risk chemotherapy agents.<br><br>There was no treatment-related mortality, no reported allergic reactions.	Access to cancer treatment
Solomons et al 2018	Rural	Counselling provided by a board-certified cancer genetic counselor and a medical oncologist via telehealth at two distant hospital-based clinics.	Pre/post-test	Fewer patients and/or others supporting their attendance were required to miss work, and childcare needs were eased slightly by tele-genetics.	Access to genetic counselling<br><br>Patient satisfaction
Thota et al 2020	Rural	Patients seen in enabled clinic rooms and undergo synchronous video-based calls with medical oncologists,	Retrospective database analysis	From 2015 to 2018, 119 patients were treated, accounting for 1,025 patient encounters. Patients saved averagely	Access to cancer treatment

		certified oncology nurses, and other subspecialists and ancillary staff located 120 miles away.		4 hours and 40 minutes and 332 miles roundtrip per encounter. Total, patients' savings were estimated to be $333,074.	
Watanabe et al 2013	Rural	Cancer patients in rural area attend local telehealth facilities, accompanied by nurses trained in symptom assessment. Ninety minutes of videoconference time for each new patient consultation, with 30 min for each follow-up visit.	Retrospective database analysis	Average per visit savings for patients seen by telehealth versus attending the cancer centre were 471.13 km, 7.96 hours, and CAD$192.71. There was high degree of satisfaction.	Access to cancer treatment<br><br>Patient satisfaction
Wagoner et al 2023	Rural	Exercise-oncology program for rural and remote communities held via ZOOM™	Quasi-experimental	Adherence to the exercise intervention was 78.2%. Fitness assessment and patient-reported outcome completion rates were above 85% pre- and post-intervention	Access to cancer treatment
**Reducing distance**					
Balata et al 2019	Low-income	Free lung health check intervention including symptom assessment, spirometry, and lung cancer risk score by nurses and annual low-dose computed tomography screening for high risk participants.	Pretest post-test experimental design	Up to 90% of those eligible attended the second screening round.	Access to screening
Charlton et al 2014	Rural	Mailing FIT, plus educational materials and survey to	Randomized controlled trial	At 6 months post intervention, 21% of the FIT group had received	Access to screening

		asymptomatic, average-risk patients overdue for screening.		CRC screening compared to 6% of the control group (P < .0001).	
Curtis et al 2018	Rural	Satellite chemotherapy infusion centres based in communities and serving as outpatient treatment locations with shared resources housed at larger academic medical centers and staffed by local and visiting clinicians.	Qualitative interviews	High-quality community hospitals were increasingly able to offer specialized oncology treatment and nursing care at greater convenience for patients.	Access to cancer treatment
van den Bruele et al 2022	Underserved women	Mobile, no-cost mammogram screening vans (mammovans)	Retrospective cohort study	32,350 women participated in the program from 2014 to 2019. There were over 4,000 mammograms performed per year.	Access to screening
**Patient Navigation**					
Menon et al 2020	Multicultural and underinsured	A community-to-clinic navigator intervention, consisting of tailored counseling calls from a trained navigator, to guide multicultural, underinsured individuals into primary care clinics to complete colorectal cancer screening.	RCT - two-phase behvioral intervention	80 participants (37.9%) in the intervention group scheduled a clinic appointment, while only 26 (19.4%) in the comparison group did. Intervention group participants were almost three times more likely to schedule a clinic appointment for screening than comparison group	Access to screening
Battaglia et al 2019	Low SES, racialized	Standards of care of navigation including: case and barrier identification, care planning to address	Randomized controlled trial	96% of participants with breast cancer and 73% of those with lung cancer initiated treatment within 90 days.	Access to cancer treatment

		barriers, and long-term tracking enhanced with legal advocates to identify and address socio-legal barriers.			
Li et al 2019	Hispanic women	A health promotion media campaign, educational outreach, a patient navigation initiative, and the provision of mammography screening services.	Mixed methods	The mammography screening rate for the target population increased from 60% to 80% throughout the program	Access to screening
Freund et al 2013	Low-income, uninsured or publicly insured, and racial minority	Navigators identifying barriers to recommended care, developing strategies to address these barriers, and tracking participants through the steps in their medical evaluation.	Randomized clinical trial	At 365 days, navigated participants had a higher proportion (89%) of those who had initiated treatment compared with control participants (87%).	Access to cancer treatment
Luckett et al. 2015	Women: Low SES, history of abuse racialized	Trained bilingual patient navigator experienced in health prevention and community health reaching out to vulnerable patients identified by the healthcare team or who self-identify as facing barriers to care.	Database analysis	No-show rates for colposcopy declined from 49.7% to 29.5% after implementation of the patient navigator program ($p < 0.0001$).	Access to screening
Percac-Lima et al 2014	Low-income, Latino and immigrant population	Culturally tailored navigation delivered by trained navigator and interpreters covering 6 other languages. Navigator educated	Randomized controlled trial	CRC screening rates improved for navigated patients (70.6% vs 58.6%, respectively $P < .001$).	Access to screening

		patients about CRC screening and explored barriers to screening.			
Ramirez et al 2014	Latino women	Culturally tailored navigation model using six bilingual Latina women aged 25 to 47 years with at least a high school diploma or college degree, and trained to coordinate care for those referred for diagnostic evaluation and treatment.	Pre- and post-test	Compared with control patients, many navigated participants initiated treatment within 30 days (69.0% versus 46.3%, P 5 .029) and 60 days (97.6% versus 73.1%, P 5 .001) after cancer diagnosis.	Access to cancer treatment
Thai et al 2022	Vietnamese-American Women	Vietnamese patient navigator who provided emotional support, education, translation, and assistance with medical bills and doctor's appointments.	Retrospective database analysis	All 96 participants attended follow-up appointments for their abnormal mammograms within 90 days. A majority of the participants reported satisfaction with their navigators.	Access to cancer treatment<br><br>Patient satisfaction
Vilchis et al 2019	Rural	Two bilingual patient navigators assisting in the coordination of health care services and provider referrals for diagnosed individuals, linking patients with community organizations/services to receive better access to high-quality health care services, and identifying barriers within the health care system and assisting	Retrospective database analysis	Intervention did not decrease time from diagnosis to treatment initiation compared to comparison group. Mean time from cancer diagnosis to treatment initiation among 41 study patients was 59.6 days and 47.1 days for non-intervention patients. There were positive reactions from patients and families.	Access to cancer treatment

		patients to overcome them.			
Williams MA et al 2022	Rural, low SES, racialized	Breast cancer nurse navigators whose works was to improve care coordination, psychosocial care, patient education, and healthcare usage.	Surveys	54 of 86 patients were navigated and strongly agreed they were prepared for the beginning of treatment as compared to non-navigated patients ($p = 0.02$).	Access to cancer treatment
Falk et al 2023	Rural	Education of women about the need for breast and cervical cancer screenings and providing navigation services to reduce financial and other barriers to Pap tests and mammograms by paying for screening, diagnostic, and follow-up clinical services.	Cross-sectional, retrospective analysis	A total of 795 (67.3%) received navigation, 494 (41.8%) received a Pap test, and 121 (10.3%) received a mammogram.	Access to screening
Watson et al 2016	Rural	A provincial standard navigation program spanning 15 isolated urban and rural ambulatory care settings in Alberta. Navigation role integrated into the existing clinical environment and involved specially trained registered nurses providing a variety of clinical supports and services including psychosocial interventions, coordination of care,	Qualitative	Navigation reduced hospital and emergency room visits, improved support for emotional and practical concerns, improved care coordination enhanced continuity in care, and provided meaningful information.	Access to treatment<br><br>Patient satisfaction

		health education, case management, and facilitation of communication between health systems and the patient.			
**Service redesign**					
Galiatsatos et al 2021	Racialized, low SES	A dedicated Tobacco Treatment Clinic (TTC), that also provided lung cancer screening for underserved groups.	Retrospective analysis	Of the 92 patients enrolled, 68 (73.9%) had lung cancer screening, with 51 patients receiving their first lung cancer screening scan through the clinic.	Access to cancer screening
Gunness et al 2023	Rural women	Establishing a breast reconstruction unit in a rural setting.	Retrospective analysis	97% of the 38 participants strongly valued having a breast reconstruction service within their community. Patients saved average of $8,478 and had a reduction of 2,800 km travel distance.	Access to cancer treatment<br><br>Patient satisfaction
Hall et al 2019	Rural and remote	Generalist occupational therapists and physiotherapists undertake compression garment, selection, fitting, and monitoring services for stabilised malignancy-related lymphoedema, supported by telehealth coaching and supervision by lymphoedema therapists.	Randomized controlled trial	Increased access to compression garment services. Ninety-two percent of patients were very satisfied with their experience.	Access to cancer treatment<br><br>Patient satisfaction
Hoskins et al 2018	Underserved African American women	Incorporating CGRA into primary care physicians' practices	Randomized controlled trial	After initiating CGRA, 112 eligible women consented to study	Access to GC

		for the systematic assessment of genetic risk of breast and/or ovarian cancer in community health clinics.		participation, and 56% of them received a referral for GC from their primary care physician. No patient was referred for GC during usual care, compared with 64% after the initiation of CGRA (P<.001).	
Dumont et al 2021	Racialized, incarcerated people	Collaboration between public health and correctional service to screen for CRC in a state prison system	Quasi-experimental	1,396 of 1,856 (75.2%) sentenced people completed an eligibility screen, and 254 of 321 (79.1%) eligible patients completed a FIT. Of the 54 patients with positive FITs, 33 (61.1%) completed follow-up colonoscopies.	Access to screening
Patel et al 2023	Low SES and racialized women	Integrating pharmacists into clinical care with providers to mitigate barriers to therapy acquisition, adherence, and toxicity management and decrease treatment delays	Quasi-experiment	A pre-intervention assessment identified an average treatment day delay of 7.7 (range, 3.2-15.3) days during the first six cycles of oral cancer. During the intervention period, 53 patients were enrolled and experienced an average treatment day delay of 2.1 (0.6-4) days, P= .0001.	Access to cancer treatment
Swayze et al 2021	Rural	Establishing a full-time Gynaecologic Oncology (GO) care in a semi-urban area. This constituted a robust support system including nurse navigation,	Retrospective cohort study	Out of 381 patients, 171 women were diagnosed prior to establishing a full-time GO office (pre-GO) and 210 after (post-GO). Post-GO patients were more likely to undergo surgery by a GO	Access to cancer treatment

		oncofertility, survivorship, palliative care, and genetic counseling.		specialist (97.1% versus 53.2%, $p < 0.01$), receive surgery locally (79.0% versus 43.3%, $p < 0.01$), and undergo complete lymph node dissection (63.3% versus 38.6%, $p < 0.01$). Patients treated with chemotherapy by GO increased from 10.3% pre-GO to 76.9% post-GO.	
Tracy et al 2013	Rural	Establishing visiting consultant clinics to provide cancer care including chemotherapy, biological treatments, and pain management services to local communities.	Retrospective database descriptive	For all rural census tracts, the median travel time fell from 51.6 to 19.2 minutes.	Access to cancer treatment
Tsai et al 2014	Racialized and low SES women	Nurse-managed health centres that provide breast health education and awareness, clinical breast screening examination, and referral for mammography. The nurse served as the initial primary care provider as well as the case manager.	Retrospective descriptive	Participant's knowledge about breast health was increased. Significant positive changes in behavior related to the early detection of breast cancer were found.	Increased knowledge<br><br>Access to screening
Wakefield et al 2023	Socio-economically deprived	A long-term palliative care unit that serves patients with a palliative diagnosis with a predicted prognosis less than 12 months. The long-	Retrospective database analysis	Within 7 years, 199 patients were admitted to the long-term unit and 98% remained there until death. All patients were offered a full holistic assessment and advance	Access to palliative care

		term unit is well integrated with surrounding community and hospital specialist palliative care teams and share joint multidisciplinary team meeting and clinical staff		care planning, with 24/7 access to specialist palliative care support. 27% of all admissions to the long-term unit were from areas in the 10% most deprived (with 41% admitted from areas in the poorest quintile).	
**Education and counselling**					
Lee-Lin et al 2013	Chinese American immigrant women	Breast health educational for participants who had not had a mammogram within the past 12 months. 1-hour group teaching conducted in both Mandarin and Cantonese, followed by an individual counseling session by phone to help overcome barriers relating to cost, procedural fears or concerns, transportation, language, child care, and health care access.	One-group pre- and post-test quasi-experimental	42 women completed the study, and 21 (50%) had a mammogram post intervention. Mean breast cancer susceptibility scores increased significantly at post-test ($t[40] = -2.88$, $p < 0.01$)	Access to screening<br><br>Increased knowledge
Lofters et al 2017	Black women	A 2-hour educational session on breast cancer myths, benefits for screening for breast and cervical cancer, meaning of screening test results and common barriers to screening, delivered to different groups of	Pre- and post-test	Increased awareness of cancer susceptibility, screening guidelines, and screening self-efficacy. 4/30 women had a mammography 2 months post training	Access to screening<br><br>Increased knowledge

		women, and experience sharing by a black woman breast cancer survivor.			
Niranjan et al 2023	Rural, poor, unemployed.	Community health advisor delivered a single educational session (approximately 30 min) on lung cancer risk, the experience of a low-dose computed tomography (LDCT) scan, and benefits and harms.	Pretest post-test	Lung cancer knowledge increased significantly from 4.64 (SD=2.37) to 7.61 (SD=2.26). 23/100 participated in lung cancer screening	Access to screening<br><br>Increased knowledge
Schroeder et al 2021	Rural	An informational poster board displayed at the oral cancer booth providing information regarding oral cancer risk factors, prevention, and signs and symptoms. Dental hygienists explain the screening process and recommended annual visual and tactile head and neck examinations. Printed educational material made available. Information regarding free and reduced cost dental clinics provided.	Cross-sectional survey	82% of participants consenting to the oral cancer screening. Tactile head and neck examination/ oral cancer screening	Access to screening<br><br>Increased knowledge
Wilson-Anderson et al 2013	Mostly African American	Educational classes regarding breast health held by faith-based groups and women's social organizations. Leaders of women's community groups	Mixed methods<br>Pre and post-test	Significant increase in knowledge: $t = -16.6$, $df = 126$, $p < .001$. 40% of participants reported yearly mammography at year 1 follow up.	Access to screening<br><br>Increased knowledge

		advertised sessions in their communities. Viewing of a Komen-produced video on breast cancer risk, breast self exam (BSE) technique, and need for mammography. Time for questions and discussion, demonstration of BSE and participants' practice of BSE.			
Williams MS et al 2022	Low socioeconomic status women	Breast health education campaign. Additional support services such as childcare, healthy cooking education, and yoga demonstration. Dissemination of health education information about breast cancer. Cancer screening with results within 2 hours.	Pre-post-surveys	Increased understanding of cervical cancer (92%), breast cancer (98%), oral cancer (81%), and health, nutrition, and exercise (77%). 57 women received a mammogram	Access to screening<br><br>Increased knowledge
Holle et al 2020	Low SES, racialized	Education and training for community pharmacists on counseling approaches for CRC patients and patient counseling with subsequent FIT.	Single group post-test	Overall, 88% of patients who chose to participate and were eligible for CRC screening sent in their FIT tests and they rated "great" satisfaction with the service	Access to screening<br><br>Increased patient satisfaction
**Financial support**					
Khalil et al. 2020	Uninsured Women	A student-run free weekly clinic operating within a large academic health centre. Patients counseled on the importance of breast cancer screening and	Retrospective chart reviews	From 2012–2017, 84.5% of the patients who obtained screening mammograms, utilized the program.	Access to screening

		offered free screening mammograms.			
Eberth et al 2018	Uninsured, or live at or below 150% poverty line.	Provision of colonoscopy screening at no cost to individuals age 50–64 years or 45 years if African Americans.	Retrospective analysis	Out of >4,000 patients referred to the program, 1,854 were eligible, 1,144 attended an in-person navigation visit, and 1,030 completed a colonoscopy.	Access to screening
Peppercorn et al 2017	Rural women	An all expenses–paid "well-women" primary care visit and elimination of copayment, coinsurance, and deductible fees for screening mammography for all women 40 years old or older.	Retrospective database analysis	Between 1998 and 2011, the annual screening rate increased from 35% to a peak of 50% among women aged 40 to 49 years and from 49% to 58% among women aged 50 to 64 years. Screening rates increased significantly ($P < .0001$) after the elimination of cost sharing.	Access to screening
Rajan et al 2015	Underserved women	Patients received a paid clinical breast examination and/or mammogram during the 12 months prior to their date of breast cancer diagnosis.	Retrospective analysis	Participants had lower rates of breast surgery (odds ratio [OR] = 0.57; 95% confidence interval [CI], 0.47-0.69), and higher rates of chemotherapy (OR = 1.15; 95% CI, 1.02-1.29) as compared with the comparison group.	Access to cancer treatment
Johnson et al 2021	Low-income, uninsured and underinsured women	A waiver was used to expand Medicaid eligibility to uninsured women screened for breast or cervical cancer within a Women's Health Check (WHC) program. Patients were assisted	Retrospective database analysis	Nine percent (9.1%) of WHC-linked cases, compared to 4.1% of non-linked cases, were diagnosed at distant stage.<br>92% of WHC-linked patients began treatment	Access to cancer treatment

		to enrol in Medicaid for breast and cervical cancer treatment.		within 60 days of diagnosis.	
**Multicomponent**					
Azizoddin, et al 2020	Veterans who were mostly African American and males of low SES.	Education of program implementers on distress screening, suicide evaluation and supportive oncology. Team consultation and design of instructional guide to encourage referrals.	Cross-sectional retrospective survey	263 veterans screened for distress in 2 years.	Access to cancer treatment
Baker, et al 2014	Latino; low income and uninsured	Mailing of fecal occult blood test (FOBT) kits with a pre-paid return envelope for home testing. Using automated reminder phone calls and text messages. Using plain language instructions and explanatory graphics to increase understanding of FOBT. Use of navigator to call patients who didn't complete the FOBT.	Randomized controlled trial	Intervention patients were much more likely than those in usual care to complete FOBT (82.2% vs 37.3%; $P < .001$) within 6 months of the date the patient was due for annual screening.	Access to screening
Davis et al. 2020	Rural	Participants received a FIT test with instructions and low literacy CRC informational pamphlet. Personal call to intervention participants and automated reminder calls to control participants if they had	Two-arm randomized controlled trial	Repeat FIT rates for CRC screening were 36.5% for those receiving the automated call and 33.6% for those receiving a personal call ($P = .30$).	Access to screening

		not returned their FIT within 4 weeks. Use of pre-addressed pre-paid envelopes to mail their FIT kits.			
Centra et al. 2023	Low SES: unhoused, marginally housed, or recent refugees.	Laminated CRC screening guides posted at each computer workstation. Laminated patient education graphics placed in waiting and examination rooms. Examination rooms stocked with FITs. FIT kits supplied to the mobile unit, for distribution to homebound and unhoused patients. Patients assisted with insurance approvals, lodging and transportation for colonoscopy procedures. Reminder letters sent to patients who failed to complete ordered colonoscopy within 3 months of referral.	Pre-test post-test intervention	CRC screening rate improved to 41.5% among patients aged 50–75. A 55% return rate was achieved with mailed FIT within 2 months**.**	Access to screening
Champion et al 2023	Women in rural Communities	Digital video disc (DVD) plus patient navigation. The DVD interactively assessed and provided messages for health beliefs. Patient navigators counseled women on	Randomized clinical trial	Intervention group had nearly twice the odds of those in the usual care group of being up to date for all screenings (odds ratio [OR], 1.84; 95% CI, 1.02-3.43; P = .048)	Access to screening

		barriers to and helped them obtain screening.			
Drake et al 2022	Socioeconomically deprived, racial minority women	A coordinated navigation approach where primary and specialty care providers helped expedite care, increase care efficiency, and standardize referral procedures across systems for all women. A mobile mammography unit deployed to serve women living in both urban and rural regions.	Retrospective analysis	During the study period, 9,789 women were enrolled in the registry for their first screening visit and 85% (n = 8289) received their care on the mobile mammography van.	Access to screening
Fang et al 2017	Korean women	A 2-hour cervical cancer educational session conducted by bilingual community health educators. Discussion of possible barriers to screening relevant to Korean American women, and follow-up and clinical management of an abnormal result. Provision of information on available low-cost or free screening sites and navigation assistance for screening. Follow-up reminder letter for screening sent 6 months after education.	Matched-pair, group-randomized design	Significantly higher cervical cancer screening rates in the 12 months after study entry compared with the control program (odds ratio [OR], 25.9; 95% confidence interval [CI], 10.1-66.1; P <.001)	Access to screening
Foley et al 2023	Rural	Development of an eLearning repository	Participatory action research	Majority of consumers accessing head and neck	Access to cancer treatment

		and rural head and neck cancer package. Video recordings for training. Mapping of care needs for all eligible patients.		cancer care locally were satisfied with the service and would recommend it.	Patient satisfaction
Hendren et al<br>2014	Low-income and uninsured patients	Letter to patient indicating they are overdue for mammography, CRC or both. Information on how uninsured patients could obtain free cancer screening. Opportunities provided for patient to call the outreach worker with questions or for assistance. Automated telephone calls with similar message as in letter and a number to call and arrange for screening.<br>Point-of-care prompts to intervention patients, to remind both the clinician and patient that the patient was past due for mammography and/or CRC screening	Randomized controlled trial	Significantly higher rates of cancer screening in intervention subjects: 29.7 % vs. 16.7 % for mammography (p=0.034) and 37.7 % vs. 16.7 % for CRC screening (p=0.0002) during the 52 weeks after randomization	Access to screening
Kim et al<br>2020	Diverse underserved, urban patient population.	Provider reminders through EMR alerts (flags) placed manually into charts of patients who might be due for CRC screening and would pop up when the provider was with the patient.	Pre-test post-test experimental design	Overall increase in CRC screenings orders completed of 21.2 percentage points	Access to screening

		Patient reminders if an order had been placed for a FIT/FOBT test 90 days ago, but the test was not yet completed. Care coordination team phoning patients if positive FITs/FOBTs were returned, to encourage them to schedule a colonoscopy. Letters mailed to patients who could not be reached by phone.			
Kiser et al 2020	Uninsured and underserved women	A patient engagement tool on cervical cancer screening, eligibility screening tool, a registration log in which to record all women enrolled in the program; and the implementation of a case log for case management.	Prospective time series	A total of 87% of women received effective care, which consisted of same-day Well Woman Health Care Program enrollment and a same-day Pap test for cervical cancer screening or an appointment to return for a well-woman visit.	Access to screening
Lane et al. 2015.	Rural	A full day program, focused on breast health education and screening via mobile mammography education. Mammography provided by mobile van at a central location. At a predetermined date, screen eligible women came to the scheduled site, took part in the	Retrospective analysis	From 2001 to 2011 over 2,300 rural women received breast health education and no cost mammography	Access to screening

		educational program, and then went on the mobile van for the mammogram.			
Le et al 2022	Low-income, uninsured or underinsured patients	Outreach and health promotion by an outreach coordinator to facilitate active referrals from community partners. Educating the community about lung cancer screening, as well as the benefits of tobacco cessation. Use of bilingual (English/Spanish) educational materials that are appropriate for a fifth-grade reading level. Navigation by nurse program managers and registered nurse navigators. Guiding patients through the lung cancer screening process in a culturally- and socioeconomically-appropriate manner.	Retrospective analysis	Of 381 ordered LDCT lung cancer screening, 334 (88%) were completed.	Access to screening
Limaye et al 2022	Male aged 65years and over	Generating a list of target patients who had not had repeat/follow-up/referral and directly contacting by telephone to invite them for a blood test. Creating patient-specific electronic pop-up reminders on the	Mixed methods	Overall increase in Prostrate cancer screening of 13.5% from baseline	Access to screening

		electronic-patient-record system for PSA follow-up/referral/repeat test. Patient education of prostate health and general self-checking, as well as benefits/risks of undergoing PSA screening in the form of patient focus groups and informative leaflets.			
Manning et al 2023	African American	Participants watched an animated video presented in 8 short chapters (M=2.95 min) on CRC. Participants completed Theory of Planned Behavior (TPB) items related to CRC screening, and items assessing CRC screening modality preference. Participants were then told that they could receive a FIT Kit free of charge. FIT Kits with return instructions for processing via a contracted laboratory mailed within one week of requests. Reminder postcards were mailed if samples were not received within 4 weeks.	Randomized controlled trial	A total of 111 participants (54.1%) requested a FIT Kit. Fifteen kits (14.4%: 8 returned following reminder) were returned for processing, yielding 1 positive finding.	Access to screening
Mayfield-Johnson et al 2016	African American women	Trained community health workers were recruited to provide	Retrospective analysis	Participants (n = 554) either received a screenings mammogram	Access to screening

		education and information at local shopping centers to inform residents on cancer risk, treatment, and resources. After the participant completed the appointment, they received a $10 gift card		(90.43%) or a diagnostic mammogram (9.57 %). Most participants (93.78%) also received a clinical breast exam**.**	
Molokwu et al 2023	Hispanic Women	Culturally tailored bilingual education, navigation activities, no-cost screening, no-cost diagnostic testing, and help finding treatment access. Navigators served as a community resource, a guide through the screening, diagnostic and treatment pathway. Navigators arranged no-cost transportation if needed	Quasi-experimental delayed intervention design	Among completers, the screening mammogram rate was 97% in the intervention group and 4.4% in the control group (RR = 22.2, 95% CI: 12.5–39.7, $p < .001$) 4 months post-intervention	Access to screening
Nnorom et al. 2021	Black and immigrant population	Provider audits: to identify barriers to screening.<br>Cancer screening education programs to increase awareness of screening disparities, gaps in provider practice, and provincial screening guidelines.<br>A patient call-back program: offering screening to all eligible patients, but not up-to-date with, breast,	Retrospective database analysis	88% of mammograms offered, 83% of FOBTs offered, and 99% of Pap tests offered were completed	Access to screening

		colorectal, or cervical cancer screening.<br>A mammography promotion day: consisting of breast cancer screening posters featuring Black and racialized people, educational sessions, breast cancer screening videos cancer screening pamphlets, and a local mammography “Just Book It” day. Also, participants were provided screening questionnaires, Afrocentric pamphlets, information on the nearest mammography site, and refreshments.			
Richman et al 2020	Uninsured and underinsured Black and Latina women	Lay breast health educators (BHE) held education and navigation sessions (community outreach) in their respective communities via a variety of community outlets (churches, grocery stores, health clinics, private homes, community centrrs, soup kitchens) to increase breast health knowledge and mammography screening among the priority population.	Pre-test post-test	Breast health knowledge was significantly higher post-intervention ($p<0.001$).<br>Almost three-quarters (72%) of those recommended/navigation to receive a mammogram, received one.	Access to screening<br><br>Increased knowledge

Sanchez-Birkhead et al 2016	Hispanic Women	Guidance, education, and emotional support to women and their families, and medical services at a free or reduced cost, without addressing legal status. Three integrated components: outreach and screening, an educational support group, and a peer home visiting program. Navigators: arrange screening, transportation, and interpretation services; arrange free or low-cost screening and travel services; follow up with providers, verify completion, and identify women diagnosed with cancer; maintain a complete database to track and call women to encourage annual re-screening; among others	Retrospective database analysis	Participants expressed feeling very satisfied with the services and believe that it addressed the specific barriers faced by this population Participation in cancer care activities	Access to psychosocial and supportive care<br><br>Patient satisfaction
Tsapatsaris & Reichman 2021	Low-income uninsured minority women	A mobile ScanVan offering free mammography screening services and patient navigation for all patients with abnormal results.	Retrospective cohort study	During the one-year study period, 17 out of 3,745 patients screened received a new diagnosis of breast cancer, corresponding to a cancer detection rate of 4.5/1,000 screened. Of the 3,745 patients screened, 258 were	Access to screening

				recalled, corresponding with a recall rate of 7%.	
Lara et al. 2018	Underserved adults	Standardization of clinic policies and workflows to make national CRC guidelines available at the point of Care. Offering FIT kits to eligible patients during nonacute visits; providing decisional support tools to educate patients regarding CRC screening options, and educating providers on national CRC screening guidelines. Establishing a CRC patient registry and a standardized process for identifying and contacting patients not up to date with CRC screening.	Retrospective analysis	CRC screening uptake increased by 18 percentage points in one health system and 10 percentage points in the second health system	Access to screening

BHE: breast health educators; BSE: breast self-exam; CRC: colorectal cancer; EMR: electronic medical record; FIT: fecal immunochemical test; FOBT: fecal occult blood test; CGRA: cancer genetic risk assessment; GC: genetic counselling; GO: Gynaecologic Oncology; LDCT: low-dose computed tomography; PSA: prostate-specific antigen; SES: socioeconomic status; WHC: Women's Health Check
