# Supplementary material for: Interventions to improve access to cancer care in underserved populations in high income countries: a systematic review
Source: Oncol Rev. 2024 Nov 5;18:1427441. doi: 10.3389/or.2024.1427441 (PMC11573526; doi:10.3389/or.2024.1427441)
Supplement: Supplementary file 2 [file Datasheet1.pdf]

Interventions to improve access to cancer care in underserved populations in high income countries: A systematic review

Appendix 1 - Search strategies for each database

Ovid MEDLINE(R) ALL <1946 to May 09, 2023>
Date of search: May 10, 2023

1 Rural Population/ 69005
2 (((rural* or remote*) adj3 (living or live? or communit* or population* or reside* or town* or village* or setting* or area*)) or "small town*" or countryside or "country side").mp. 148091
3 disabled persons/ or persons with hearing impairments/ or visually impaired persons/ 52945
4 ((learning or intellectual* or physical* or mobility or developmental* or multiple) adj2 (disabl* or disabilit*)).mp. 104398
5 ("hearing impair*" or deaf* or "hard of hearing" or "hearing los*").mp. 120436
6 brain damage, chronic/ or brain injury, chronic/ or cerebral palsy/ 37287
7 cerebral palsy.mp. 31764
8 ("brain damage*" or "brain* injur*").mp. 138461
9 exp Autism Spectrum Disorder/ 41741
10 (autism or autistic* or Asperger*).mp. 69375
11 exp hearing loss/ or blindness/ or exp deaf-blind disorders/ 97983
12 (blind* or "visual* impair*" or "vision los*").mp. 439407
13 self-help devices/ or communication aids for disabled/ or exp wheelchairs/ 13168
14 canes/ or crutches/ or walkers/ 1760
15 Hearing Aids/ 9898
16 Cochlear Implants/ 12517
17 (wheelchair* or crutch* or cane? or walker* or "mobility aid*" or "hearing aid*" or "cochlear implant*" or "vision aid*").mp. 65830
18 (dyslexi* or dysgraphi* or dyspraxi* or dyscalculi*).mp. 12487
19 (((people* or person* or individual* or m?n or wom?n or population*) adj3 (disabl* or disabilit*)) or handicapped).mp. 75380
20 Vulnerable Populations/ 12763
21 low socioeconomic status/ 27
22 ((disadvantage* or vulnerab* or raciali* or poor* or "low* income*" or socioeconomic* or socio-economic* or discriminat* or marginali* or underserved or underrepresented) adj3 (population* or group* or communit* or people* or person* or m?n or wom?n or male* or female* or adult* or subpopulation or sub-population* or patient*)).mp. 245709
23 asian/ or "black or african american"/ 68279
24 exp "Hispanic or Latino"/ 37956
25 exp "emigrants and immigrants"/ or refugees/ 27615
26 Ethnic and Racial Minorities/ 503

27 (black? or african* or afro* or latin? or non-white or immigrant* or emigrant* or refugee* or "asylum seek*" or asian or non-caucasian or "racial minorit*" or "ethnic minorit*").mp. 638562
28 exp "Sexual and Gender Minorities"/ 15838
29 bisexuality/ or exp homosexuality/ or transsexualism/ 39315
30 (LGB* or homosexual* or gay? or lesbian* or bisexual* or transgender* or transsexual* or queer* or "men who have sex with men" or MSM or "women who have sex with women" or WSW or "gender minorit*" or "sex* minorit*").mp. 70576
31 Social Determinants of Health/ 6314
32 Social determinants of health.mp. 14332
33 or/1-32 2004689
34 exp neoplasms/dt, th, rt, su or exp neoplasms by histologic type/dt, th, rt, su or exp neoplasms by site/dt, th, rt, su 1630616
35 (exp neoplasms/ or exp neoplasms by histologic type/ or exp neoplasms by site/) and (treatment* or care or caring or management or therap* or medicine* or drug* or surger* or surgical).ab. 1589138
36 ((Cancer* or Neoplasm* or malignan* or tumor* or tumour*) adj7 (treat* or care or caring or management or therap* or medicine* or drug* or surger* or surgical)).mp. 1281036
37 exp Antineoplastic Agents/ 1239650
38 exp radiotherapy/ or exp chemoradiotherapy/ 206540
39 (chemotherap* or radiotherap* or chemoradiotherap*).mp. 866915
40 oncological.mp. 33951
41 ((leukemia or sarcoma* or lymphoma* or myeloma* or melanoma* or carcinoma*) adj7 (treat* or care or caring or management or therap* or medicine* or drug* or surger* or surgical)).mp. 308643
42 or/34-41 3417474
43 Health Services Accessibility/ 85195
44 ((health or healthcare or medicine* or therap* or treatment* or program* or service*) adj4 (access* or availab* or convenien* or delay* or equal* or equit* or unequal* or inequal* or barrier* or obstacle*)).mp. 384930
45 access to care*.mp. 17416
46 universal access*.mp. 2542
47 or/43-46 393076
48 exp Canada/ 180818
49 (Canad* or "british columbia*" or alberta* or saskatchewan or manitoba* or ontario or ontarian* or quebec or "nova scotia*" or "prince edward island*" or "new brunswick" or newfoundland or yukon or "northwest territor*" or NWT or nunavut).mp. 271214
50 exp Australia/ 169200
51 (Australia* or queensland or "new south wales" or tasmania).mp. 231161
52 New Zealand/ 44020
53 ("New Zealand*" or auckland or "hawkes bay" or gisborne or taranaki or wellington or canterbury or marlborough or nelson or westland or otago or southland).mp. 87096
54 exp united kingdom/ or exp england/ or exp scotland/ 389439
55 ("United kingdom" or UK or england or scotland or wales or ireland).mp. 541829

56 exp "scandinavian and nordic countries"/ or exp denmark/ or exp norway/ 220244
57 (Scandinavia* or Norway or Norwegian* or Sweden or swedish or denmark or finland or Iceland or nordic*).mp. 305949
58 exp united states/ or exp appalachian region/ or exp great lakes region/ or exp mid-atlantic region/ or exp midwestern united states/ or exp new england/ or exp northwestern united states/ or exp pacific states/ or exp southeastern united states/ or exp southwestern united states/ 1458249
59 ("united states" or USA or alabama or alaska or arizona or arkansas or california or colorado or connecticut or delaware or florida or georgia or hawaii or idaho or illinois or indiana or iowa or kansas or kentucky or louisiana or maine or maryland or massachusetts or michigan or minnesota or mississippi or missouri or montana or nebraska or nevada or "new hampshire" or "new jersey" or "new mexico" or "new york" or "north carolina" or "north dakota" or ohio or oklahoma or oregon or pennsylvania or "rhode island" or "south carolina" or "south dakota" or tennessee or texas or utah or vermont or virginia or washington or "west virginia" or wisconsin or wyoming).ti,ab. 818655
60 or/48-59 3170446
61 33 and 42 and 47 and 60 3180
62 limit 61 to (case reports or comment or editorial or letter or news) 67
63 61 not 62 3113
64 limit 63 to english language 3107
65 limit 64 to yr="2013 -Current" 1921

OVID Embase <1974 to 2023 May 09>
Date of search: May 10, 2023

1 rural population/ 55882
2 (((rural* or remote*) adj3 (living or live? or communit* or population* or reside* or town* or village* or setting* or area*)) or "small town*" or countryside or "country side").mp. 189900
3 disabled person/ or mentally disabled person/ or exp physically disabled person/ 50446
4 ((learning or intellectual* or physical* or mobility or developmental* or multiple) adj2 (disabl* or disabilit*)).mp. 84266
5 ("hearing impair*" or deaf* or "hard of hearing" or "hearing los*").mp. 156481
6 exp disability/ 189533
7 cerebral palsy/ 44182
8 chronic brain disease/ 3283
9 brain injury/ or brain damage/ 136046
10 ("brain damage*" or "brain* injur*").mp. 239563
11 exp autism/ 93920
12 (autism or autistic* or Asperger*).mp. 103105
13 hearing impairment/ or deafblindness/ 70837
14 blindness/ 40281
15 (blind* or "visual* impair*" or "vision los*").mp. 657781

16 communication aid/ 2697
17 rehabilitation equipment/ or assistive scooter/ or blind mobility aid/ or dysphagia aid/ or exp "exoskeleton (rehabilitation)"/ or learning aid/ or sensory aid/ or exp walking aid/ or exp wheelchair/ 27927
18 hearing aid/ or exp cochlea prosthesis/ or digital hearing aid/ 31777
19 (wheelchair* or crutch* or cane? or walker* or "mobility aid*" or "hearing aid*" or "cochlear implant*" or "vision aid*").mp. 84919
20 (dyslexi* or dysgraphi* or dyspraxi* or dyscalculi*).mp. 15725
21 (((people* or person* or individual* or m?n or wom?n or population*) adj3 (disabl* or disabilit*)) or handicapped).mp. 83284
22 exp vulnerable population/ 27285
23 exp lowest income group/ 35755
24 ((disadvantage* or vulnerab* or raciali* or poor* or "low* income*" or socioeconomic* or socio-economic* or discriminat* or marginali* or underserved or underrepresented) adj3 (population* or group* or communit* or people* or person* or m?n or wom?n or male* or female* or adult* or subpopulation or sub-population* or patient*)).mp. 373030
25 asian american/ or asian continental ancestry group/ or exp black person/ or british asian/ or exp hispanic/ or exp migrant/ or exp multiracial person/ 259407
26 exp forced migrant/ or exp refugee/ 17408
27 ethnic group/ 78785
28 (black? or african* or afro* or latin? or non-white or immigrant* or emigrant* or refugee* or "asylum seek*" or asian or non-caucasian or "racial minorit*" or "ethnic minorit*").mp. 796143
29 exp "sexual and gender minority"/ or exp lgbtqia+ people/ 39216
30 exp lgbt people/ or exp "transgender and gender nonbinary"/ 18788
31 (LGB* or homosexual* or gay? or lesbian* or bisexual* or transgender* or transsexual* or queer* or "men who have sex with men" or MSM or "women who have sex with women" or WSW or "gender minorit*" or "sex* minorit*").mp. 90794
32 social determinants of health/ 18805
33 Social determinants of health.mp. 23782
34 or/1-33 2896361
35 exp neoplasm/dt, rt, su, th [Drug Therapy, Radiotherapy, Surgery, Therapy] 1633414
36 exp neoplasm/ and (treatment* or care or caring or management or therap* or medicine* or drug* or surger* or surgical).ab. 2910776
37 ((Cancer* or Neoplasm* or malignan* or tumor* or tumour*) adj7 (treat* or care or caring or management or therap* or medicine* or drug* or surger* or surgical)).mp. 2294042
38 exp antineoplastic agent/ 2752411
39 chemoradiotherapy/ 67989
40 cancer therapy/ or exp cancer chemotherapy/ or exp cancer radiotherapy/ 877684
41 (chemotherap* or radiotherap* or chemoradiotherap*).mp. 1501706
42 oncological.mp. 67266

43 ((leukemia or sarcoma* or lymphoma* or myeloma* or melanoma* or carcinoma*) adj7 (treat* or care or caring or management or therap* or medicine* or drug* or surger* or surgical)).mp. 583805
44 or/35-43 5652766
45 health care access/ or unmet medical need/ 86049
46 ((health or healthcare or medicine* or therap* or treatment* or program* or service*) adj4 (access* or availab* or convenien* or delay* or equal* or equit* or unequal* or inequal* or barrier* or obstacle*)).mp. 544992
47 access to care*.mp. 24710
48 universal access*.mp. 3142
49 45 or 46 or 47 or 48 559989
50 exp Canada/ 214521
51 (Canad* or "british columbia*" or alberta* or saskatchewan or manitoba* or ontario or ontarian* or quebec or "nova scotia*" or "prince edward island*" or "new brunswick" or newfoundland or yukon or "northwest territor*" or NWT or nunavut).mp. 374596
52 australia and new zealand/ or exp australia/ or new zealand/ 262153
53 (Australia* or queensland or "new south wales" or tasmania).mp. 319767
54 ("New Zealand*" or auckland or "hawkes bay" or gisborne or taranaki or wellington or canterbury or marlborough or nelson or westland or otago or southland).mp. 135306
55 exp united kingdom/ or exp great britain/ 461654
56 ("United kingdom" or UK or england or scotland or wales or ireland).mp. 1005599
57 exp scandinavia/ or exp finland/ or exp norway/ 231455
58 (Scandinavia* or Norway or Norwegian* or Sweden or swedish or denmark or finland or Iceland or nordic*).mp. 404821
59 exp United States/ 1366950
60 ("united states" or USA or alabama or alaska or arizona or arkansas or california or colorado or connecticut or delaware or florida or georgia or hawaii or idaho or illinois or indiana or iowa or kansas or kentucky or louisiana or maine or maryland or massachusetts or michigan or minnesota or mississippi or missouri or montana or nebraska or nevada or "new hampshire" or "new jersey" or "new mexico" or "new york" or "north carolina" or "north dakota" or ohio or oklahoma or oregon or pennsylvania or "rhode island" or "south carolina" or "south dakota" or tennessee or texas or utah or vermont or virginia or washington or "west virginia" or wisconsin or wyoming).ti,ab. 1148810
61 or/50-60 3932057
62 34 and 44 and 49 and 61 5896
63 limit 62 to (books or chapter or conference abstract or conference paper or "conference review" or editorial or letter or note) 2624
64 62 not 63 3272
65 limit 64 to (english language and yr="2013 -Current") 2149

OVID APA PsycInfo <1806 to May Week 2 2023>
Date of search: May 10, 2023

1 rural environments/ or rural health/ 22225
2 (((rural* or remote*) adj3 (living or live? or communit* or population* or reside* or town* or village* or setting* or area*)) or "small town*" or countryside or "country side").mp. 33326
3 disabilities/ or exp learning disabilities/ or exp multiple disabilities/ or exp reading disabilities/ 58274
4 hearing loss/ or exp deafness/ 16991
5 exp blindness/ 5954
6 ((learning or intellectual* or physical* or mobility or developmental* or multiple) adj2 (disabl* or disabilit*)).mp. 71499
7 ("hearing impair*" or deaf* or "hard of hearing" or "hearing los*").mp. 32883
8 cerebral palsy/ or brain disorders/ 11582
9 cerebral palsy.mp. 9190
10 ("brain damage*" or "brain* injur*").mp. 57883
11 autism spectrum disorders/ 54044
12 (autism or autistic* or Asperger*).mp. 68935
13 (blind* or "visual* impair*" or "vision los*").mp. 73721
14 mobility aids/ 1287
15 exp hearing aids/ 5072
16 (wheelchair* or crutch* or cane? or walker* or "mobility aid*" or "hearing aid*" or "cochlear implant*" or "vision aid*").mp. 14927
17 (dyslexi* or dysgraphi* or dyspraxi* or dyscalculi*).mp. 14152
18 (((people* or person* or individual* or m?n or wom?n or population*) adj3 (disabl* or disabilit*)) or handicapped).mp. 54653
19 ((disadvantage* or vulnerab* or raciali* or poor* or "low* income*" or socioeconomic* or socio-economic* or discriminat* or marginali* or underserved or underrepresented) adj3 (population* or group* or communit* or people* or person* or m?n or wom?n or male* or female* or adult* or subpopulation or sub-population* or patient*)).mp. 85743
20 asians/ or exp japanese cultural groups/ or exp southeast asian cultural groups/ 15594
21 blacks/ 59238
22 exp "latinos/latinas"/ 33417
23 exp immigration/ or refugees/ 33366
24 exp minority groups/ 20535
25 (black? or african* or afro* or latin? or non-white or immigrant* or emigrant* or refugee* or "asylum seek*" or asian or non-caucasian or "racial minorit*" or "ethnic minorit*").mp. 245679
26 exp lgbtq/ or exp homosexuality/ or exp intersex/ 39686
27 (LGB* or homosexual* or gay? or lesbian* or bisexual* or transgender* or transsexual* or queer* or "men who have sex with men" or MSM or "women who have sex with women" or WSW or "gender minorit*" or "sex* minorit*").mp. 64447
28 social determinants of health.mp. 3254
29 or/1-28 773778
30 exp neoplasms/ 60543
31 ((Cancer* or Neoplasm* or malignan* or tumor* or tumour*) adj7 (treat* or care or caring or management or therap* or medicine* or drug* or surger* or surgical)).mp. 36821

32 antineoplastic drugs/ 320
33 chemotherapy/ 3574
34 radiation therapy/ 1444
35 (chemotherap* or radiotherap* or chemoradiotherap*).mp. 9298
36 oncological.mp. 1059
37 ((leukemia or sarcoma* or lymphoma* or myeloma* or melanoma* or carcinoma*) adj7 (treat* or care or caring or management or therap* or medicine* or drug* or surger* or surgical)).mp. 1604
38 or/30-37 70927
39 health care access/ 2346
40 ((health or healthcare or medicine* or therap* or treatment* or program* or service*) adj4 (access* or availab* or convenien* or delay* or equal* or equit* or unequal* or inequal* or barrier* or obstacle*)).mp. 94251
41 access to care*.mp. 4837
42 universal access*.mp. 504
43 39 or 40 or 41 or 42 96470
44 (Canad* or "british columbia*" or alberta* or saskatchewan or manitoba* or ontario or ontarian* or quebec or "nova scotia*" or "prince edward island*" or "new brunswick" or newfoundland or yukon or "northwest territor*" or NWT or nunavut).mp. 71854
45 (Australia* or queensland or "new south wales" or tasmania).mp. 65574
46 ("New Zealand*" or auckland or "hawkes bay" or gisborne or taranaki or wellington or canterbury or marlborough or nelson or westland or otago or southland).mp. 18862
47 ("United kingdom" or UK or england or scotland or wales or ireland).mp. 104370
48 (Scandinavia* or Norway or Norwegian* or Sweden or swedish or denmark or finland or Iceland or nordic*).mp. 66484
49 ("united states" or USA or alabama or alaska or arizona or arkansas or california or colorado or connecticut or delaware or florida or georgia or hawaii or idaho or illinois or indiana or iowa or kansas or kentucky or louisiana or maine or maryland or massachusetts or michigan or minnesota or mississippi or missouri or montana or nebraska or nevada or "new hampshire" or "new jersey" or "new mexico" or "new york" or "north carolina" or "north dakota" or ohio or oklahoma or oregon or pennsylvania or "rhode island" or "south carolina" or "south dakota" or tennessee or texas or utah or vermont or virginia or washington or "west virginia" or wisconsin or wyoming).ti,ab. 316505
50 44 or 45 or 46 or 47 or 48 or 49 603078
51 29 and 38 and 43 and 50 550
52 limit 51 to (english language and yr="2013 -Current") 310

CINAHL via EBSCOhost (1936 - Present)
Date of search: May 10, 2023

S1 (MH "Rural Population") 12,859
S2 (((rural* or remote*) N3 (living or live# or communit* or population* or reside* or town* or village* or setting* or area*)) or "small town*" or countryside or "country side") 62,414

S3 (MH "Persons with Disabilities") 37,375
S4 (MH "Deafness") OR (MH "Deaf-Blind Disorders+") 8,513
S5 (MH "Blindness+") 6,753
S6 ((learning or intellectual* or physical* or mobility or developmental* or multiple) N2 (disabl* or disabilit*)) 46,363
S7 ("hearing impair*" or deaf* or "hard of hearing" or "hearing los*") 39,818
S8 (MH "Brain Damage, Chronic+") 14,829
S9 "cerebral palsy" OR (("brain damage*" or "brain* injur*")) 58,534
S10 (MH "Asperger Syndrome") OR (MH "Autistic Disorder") 29,322
S11 (autism or autistic* or Asperger*) 37,463
S12 (blind* or "visual* impair*" or "vision los*") 136,070
S13 (MH "Ambulation Aids+") OR (MH "Communication Aids for Persons with Disabilities+") OR (MH "Cochlear Implant+") OR (MH "Hearing Aids+") OR (MH "Wheelchairs+") 30,773
S14 (wheelchair* or crutch* or cane# or walker* or "mobility aid*" or "hearing aid*" or "cochlear implant*" or "vision aid*") 58,874
S15 (dyslexi* or dysgraphi* or dyspraxi* or dyscalculi*) 3,697
S16 (((people* or person* or individual* or m#n or wom#n or population*) N3 (disabl* or disabilit*)) or handicapped) 71,735
S17 (MH "Special Populations") 8,235
S18 ((disadvantage* or vulnerab* or raciali* or poor* or "low* income*" or socioeconomic* or socio-economic* or discriminat* or marginali* or underserved or underrepresented) N3 (population* or group* or communit* or people* or person* or m#n or wom#n or male* or female* or adult* or subpopulation or sub-population* or patient*)) 107,140
S19 (MH "Black Persons+") OR (MH "Asians+") OR (MH "Hispanic Americans+") 108,996
S20 (MH "Immigrants+") OR (MH "Refugees+") 25,925
S21 (MH "Emigration and Immigration") OR (MH "Transients and Migrants") 12,930
S22 (black# or african* or afro* or latin? or non-white or immigrant* or emigrant* or refugee* or "asylum seek*" or asian or non-caucasian or "racial minorit*" or "ethnic minorit*") 214,424
S23 (MH "Sexual and Gender Minorities+") OR (MH "Gender-Nonconforming Persons+") OR (MH "LGBTQ+ Persons+") OR (MH "Gay Persons+") OR (MH "Transgender Persons+") 19,275
S24 (MH "Homosexuality") OR (MH "Bisexuality") OR (MH "Asexuality") OR (MH "Questioning Persons") 8,064
S25 (LGB* or homosexual* or gay# or lesbian* or bisexual* or transgender* or transsexual* or queer* or "men who have sex with men" or MSM or "women who have sex with women" or WSW or "gender minorit*" or "sex* minorit*") 39,736
S26 (MH "Social Determinants of Health") 10,622
S27 "Social determinants of health" 13,345
S28 S1 OR S2 OR S3 OR S4 OR S5 OR S6 OR S7 OR S8 OR S9 OR S10 OR S11 OR S12 OR S13 OR S14 OR S15 OR S16 OR S17 OR S18 OR S19 OR S20 OR S21 OR S22 OR S23 OR S24 OR S25 OR S26 OR S27 818,849

S29 (MH "Neoplasms+/DT/RT/SU/TH") OR (MH "Neoplasms by Histologic Type+/DT/RA/SU/TH") OR (MH "Neoplasms by Site+/DT/RA/SU/TH") 317,436
S30 ((Cancer* or Neoplasm* or malignan* or tumor* or tumour*) N7 (treat* or care or caring or management or therap* or medicine* or drug* or surger* or surgical)) 378,695
S31 (MH "Antineoplastic Agents+") 137,556
S32 (MH "Chemotherapy, Cancer+") OR (MH "Chemoradiotherapy+") 38,058
S33 (MH "Radiotherapy") 20,317
S34 (chemotherap* or radiotherap* or chemoradiotherap*) 161,602
S35 oncological 6,814
S36 ((leukemia or sarcoma* or lymphoma* or myeloma* or melanoma* or carcinoma*) N7 (treat* or care or caring or management or therap* or medicine* or drug* or surger* or surgical)) 104,757
S37 S29 OR S30 OR S31 OR S32 OR S33 OR S34 OR S35 OR S36 539,824
S38 (MH "Health Services Accessibility+") OR (MH "Healthcare Disparities") 116,888
S39 ((health or healthcare or medicine* or therap* or treatment* or program* or service*) N4 (access* or availab* or convenien* or delay* or equal* or equit* or unequal* or inequal* or barrier* or obstacle*)) 232,767
S40 "access to care*" 7,603
S41 "universal access*" 1,127
S42 S38 OR S39 OR S40 OR S41 246,557
S43 (MH "Canada+") 111,484
S44 (Canad* or "british columbia*" or alberta* or saskatchewan or manitoba* or ontario or ontarian* or quebec or "nova scotia*" or "prince edward island*" or "new brunswick" or newfoundland or yukon or "northwest territor*" or NWT or nunavut) 155,045
S45 (MH "Australia+") 127,808
S46 (Australia* or queensland or "new south wales" or tasmania) 156,740
S47 (MH "New Zealand") 31,679
S48 ("New Zealand*" or auckland or "hawkes bay" or gisborne or taranaki or wellington or canterbury or marlborough or nelson or westland or otago or southland) 67,580
S49 (MH "United Kingdom+") OR (MH "Great Britain+") 326,966
S50 ("United kingdom" or UK or england or scotland or wales or ireland) 406,209
S51 (MH "Scandinavia+") 81,110
S52 (Scandinavia* or Norway or Norwegian* or Sweden or swedish or denmark or finland or Iceland or nordic*) 104,370
S53 (MH "United States+") OR (MH "United States by Individual State+") OR (MH "United States by Region+") 746,688
S54 TI (("united states" or USA or alabama or alaska or arizona or arkansas or california or colorado or connecticut or delaware or florida or georgia or hawaii or idaho or illinois or indiana or iowa or kansas or kentucky or louisiana or maine or maryland or massachusetts or michigan or minnesota or mississippi or missouri or montana or nebraska or nevada or "new hampshire" or "new jersey" or "new mexico" or "new york" or "north carolina" or "north dakota" or ohio or oklahoma or oregon or pennsylvania or "rhode island" or "south carolina" or "south dakota" or

tennessee or texas or utah or vermont or virginia or washington or "west virginia" or wisconsin or wyoming)) OR AB (("united states" or USA or alabama or alaska or arizona or arkansas or california or colorado or connecticut or delaware or florida or georgia or hawaii or idaho or illinois or indiana or iowa or kansas or kentucky or louisiana or maine or maryland or massachusetts or michigan or minnesota or mississippi or missouri or montana or nebraska or nevada or "new hampshire" or "new jersey" or "new mexico" or "new york" or "north carolina" or "north dakota" or ohio or oklahoma or oregon or pennsylvania or "rhode island" or "south carolina" or "south dakota" or tennessee or texas or utah or vermont or virginia or washington or "west virginia" or wisconsin or wyoming)) 452,525

S55 S43 OR S44 OR S45 OR S46 OR S47 OR S48 OR S49 OR S50 OR S51 OR S52 OR S53 OR S54 1,778,336

S56 S28 AND S37 AND S42 AND S55 1,879

S57 Limit S56 to English language, publication date 2013-present, scholarly (peer-reviewed) journals 1,194

Scopus (1976 - Present)
Date of search: May 10, 2023

((TITLE-ABS-KEY ((canad* OR "british columbia*" OR alberta* OR saskatchewan OR manitoba* OR ontario OR ontarian* OR quebec OR "nova scotia*" OR "prince edward island*" OR "new brunswick" OR newfoundland OR yukon OR "northwest territor*" OR nwt OR nunavut)) OR TITLE-ABS-KEY ((australia* OR queensland OR "new south wales" OR tasmania)) OR TITLE-ABS-KEY (("New Zealand*" OR auckland OR "hawkes bay" OR gisborne OR taranaki OR wellington OR canterbury OR marlborough OR nelson OR westland OR otago OR southland)) OR TITLE-ABS-KEY (("United kingdom" OR uk OR england OR scotland OR wales OR ireland)) OR TITLE-ABS-KEY ((scandinavia* OR norway OR norwegian* OR sweden OR swedish OR denmark OR finland OR iceland OR nordic*)) OR TITLE-ABS-KEY (("united states" OR usa OR alabama OR alaska OR arizona OR arkansas OR california OR colorado OR connecticut OR delaware OR florida OR georgia OR hawaii OR idaho OR illinois OR indiana OR iowa OR kansas OR kentucky OR louisiana OR maine OR maryland OR massachusetts OR michigan OR minnesota OR mississippi OR missouri OR montana OR nebraska OR nevada OR "new hampshire" OR "new jersey" OR "new mexico" OR "new york" OR "north carolina" OR "north dakota" OR ohio OR oklahoma OR oregon OR pennsylvania OR "rhode island" OR "south carolina" OR "south dakota" OR tennessee OR texas OR utah OR vermont OR virginia OR washington OR "west virginia" OR wisconsin OR wyoming)))) AND ((TITLE-ABS-KEY (((health OR healthcare OR medicine* OR therap* OR treatment* OR program* OR service*) W/4 (access* OR availab* OR convenien* OR delay* OR equal* OR equit* OR unequal* OR inequal* OR barrier* OR obstacle*))) OR TITLE-ABS-KEY ("access to care*" OR "universal access*"))) AND ((TITLE-ABS-KEY (((cancer* OR neoplasm* OR malignan* OR tumor* OR tumour*) W/7 (treat* OR care OR caring OR management OR therap* OR medicine* OR drug* OR surger* OR surgical))) OR TITLE-ABS-KEY ((chemotherap* OR radiotherap* OR

chemoradiotherap*)) OR TITLE-ABS-KEY (oncological) OR TITLE-ABS-KEY (((leukemia OR sarcoma* OR lymphoma* OR myeloma* OR melanoma* OR carcinoma*) W/7 (treat* OR care OR caring OR management OR therap* OR medicine* OR drug* OR surger* OR surgical))))) AND ((TITLE-ABS-KEY (((rural* OR remote*) W/3 (living OR live? OR communit* OR population* OR reside* OR town* OR village* OR setting* OR area*)) OR "small town*" OR countryside OR "country side")) OR (TITLE-ABS-KEY ((learning OR intellectual* OR physical* OR mobility OR developmental* OR multiple) W/2 (disabl* OR disabilit*))) OR ((TITLE-ABS-KEY (("hearing impair*" OR deaf* OR "hard of hearing" OR "hearing los*")) OR TITLE-ABS-KEY ("cerebral palsy" OR "brain damage*" OR "brain* injur*") OR TITLE-ABS-KEY ((autism OR autistic* OR asperger*)) OR TITLE-ABS-KEY ((blind* OR "visual* impair*" OR "vision los*")) OR TITLE-ABS-KEY ((wheelchair* OR crutch* OR cane? OR walker* OR "mobility aid*" OR "hearing aid*" OR "cochlear implant*" OR "vision aid*")) OR TITLE-ABS-KEY ((dyslexi* OR dysgraphi* OR dyspraxi* OR dyscalculi*)))) OR ((TITLE-ABS-KEY ((((people* OR person* OR individual* OR m?n OR wom?n OR population*) W/3 (disabl* OR disabilit*)) OR handicapped)) OR TITLE-ABS-KEY (((disadvantage* OR vulnerab* OR raciali* OR poor* OR "low* income*" OR socioeconomic* OR socio-economic* OR discriminat* OR marginali* OR underserved OR underrepresented) W/3 (population* OR group* OR communit* OR people* OR person* OR m?n OR wom?n OR male* OR female* OR adult* OR subpopulation OR sub-population* OR patient*))) OR TITLE-ABS-KEY ((black? OR african* OR afro* OR latin? OR non-white OR immigrant* OR emigrant* OR refugee* OR "asylum seek*" OR asian OR non-caucasian OR "racial minorit*" OR "ethnic minorit*")) OR TITLE-ABS-KEY ((lgb* OR homosexual* OR gay? OR lesbian* OR bisexual* OR transgender* OR transsexual* OR queer* OR "men who have sex with men" OR msm OR "women who have sex with women" OR wsw OR "gender minorit*" OR "sex* minorit*")) OR TITLE-ABS-KEY ("Social determinants of health"))))) AND (LIMIT-TO (PUBYEAR , 2023) OR LIMIT-TO (PUBYEAR , 2022) OR LIMIT-TO (PUBYEAR , 2021) OR LIMIT-TO (PUBYEAR , 2020) OR LIMIT-TO (PUBYEAR , 2019) OR LIMIT-TO (PUBYEAR , 2018) OR LIMIT-TO (PUBYEAR , 2017) OR LIMIT-TO (PUBYEAR , 2016) OR LIMIT-TO (PUBYEAR , 2015) OR LIMIT-TO (PUBYEAR , 2014) OR LIMIT-TO (PUBYEAR , 2013)) AND (LIMIT-TO (DOCTYPE , "ar") OR LIMIT-TO (DOCTYPE , "re")) AND (LIMIT-TO (LANGUAGE , "English")) Results 1755

Cochrane Library via Wiley (1993 - Present)
Date of search: May 10, 2023

#1 (((rural* or remote*) NEAR/3 (living or live? or communit* or population* or reside* or town* or village* or setting* or area*)) or (small NEXT town*) or countryside or "country side"):ti,ab,kw 8118
#2 ((learning or intellectual* or physical* or mobility or developmental* or multiple) NEAR/2 (disabl* or disabilit*)):ti,ab,kw 6394
#3 ((hearing NEXT impair*) or deaf* or "hard of hearing" or "hearing loss"):ti,ab,kw 5105
#4 ("cerebral palsy" or (brain NEXT (damag* or injur*))):ti,ab,kw 13299

#5 (autism or autistic* or Asperger*):ti,ab,kw 4978
#6 (blind* or (visual* NEXT impair*) or "vision loss"):ti,ab,kw 442067
#7 (wheelchair* or crutch* or cane? or walker* or (mobility NEXT aid*) or (hearing NEXT aid*) or (cochlear NEXT implant*) or (vision NEXT aid*)):ti,ab,kw 4180
#8 (dyslexi* or dysgraphi* or dyspraxi* or dyscalculi*):ti,ab,kw 644
#9 (((people* or person* or individual* or m?n or wom?n or population*) NEAR/3 (disabl* or disabilit*)) or handicapped):ti,ab,kw 3848
#10 ((disadvantage* or vulnerab* or raciali* or poor* or "low* income*" or socioeconomic* or socio-economic* or discriminat* or marginali* or underserved or underrepresented) NEAR/3 (population* or group* or communit* or people* or person* or m?n or wom?n or male* or female* or adult* or subpopulation or sub-population* or patient*)):ti,ab,kw 21100
#11 (black? or african* or afro* or latin? or non-white or immigrant* or emigrant* or refugee* or (asylum NEXT seek*) or asian or non-caucasian or ((racial or ethnic) NEXT minorit*)):ti,ab,kw 39451
#12 (LGB* or homosexual* or gay? or lesbian* or bisexual* or transgender* or transsexual* or queer* or "men who have sex with men" or MSM or "women who have sex with women" or WSW or ((gender or sex*) NEXT minorit*)):ti,ab,kw 3132
#13 Social determinants of health:ti,ab,kw 460
#14 {or #1-#13} 522785
#15 ((Cancer* or Neoplasm* or malignan* or tumor* or tumour*) NEAR/7 (treat* or care or caring or management or therap* or medicine* or drug* or surger* or surgical)):ti,ab,kw 148512
#16 (chemotherap* or radiotherap* or chemoradiotherap*):ti,ab,kw 113822
#17 oncological:ti,ab,kw 2711
#18 ((leukemia or sarcoma* or lymphoma* or myeloma* or melanoma* or carcinoma*) NEAR/7 (treat* or care or caring or management or therap* or medicine* or drug* or surger* or surgical)):ti,ab,kw 48488
#19 {or #15-#18} 203924
#20 ((health or healthcare or medicine* or therap* or treatment* or program* or service*) NEAR/4 (access* or availab* or convenien* or delay* or equal* or equit* or unequal* or inequal* or barrier* or obstacle*)):ti,ab,kw 36275
#21 access to care:ti,ab,kw 780
#22 universal access:ti,ab,kw 79
#23 #20 or #21 or #22 36711
#24 (Canad* or "british columbia" or alberta* or saskatchewan or manitoba* or ontario or ontarian* or quebec or "nova scotia" or "prince edward island" or "new brunswick" or newfoundland or yukon or "northwest territory" or NWT or nunavut):ti,ab,kw 25065
#25 (Australia* or queensland or "new south wales" or tasmania):ti,ab,kw 21523
#26 ("New Zealand" or auckland or "hawkes bay" or gisborne or taranaki or wellington or canterbury or marlborough or nelson or westland or otago or southland):ti,ab,kw 9268
#27 ("United kingdom" or UK or england or scotland or wales or ireland):ti,ab,kw 38048
#28 (Scandinavia* or Norway or Norwegian* or Sweden or swedish or denmark or finland or Iceland or nordic*):ti,ab,kw 24767

#29 ("united states" or USA or alabama or alaska or arizona or arkansas or california or colorado or connecticut or delaware or florida or georgia or hawaii or idaho or illinois or indiana or iowa or kansas or kentucky or louisiana or maine or maryland or massachusetts or michigan or minnesota or mississippi or missouri or montana or nebraska or nevada or "new hampshire" or "new jersey" or "new mexico" or "new york" or "north carolina" or "north dakota" or ohio or oklahoma or oregon or pennsylvania or "rhode island" or "south carolina" or "south dakota" or tennessee or texas or utah or vermont or virginia or washington or "west virginia" or wisconsin or wyoming):ti,ab,kw 90912

#30 #24 or #25 or #26 or #27 or #28 or #29 189130

#31 #14 and #19 and #23 and #30 220
